# Proton-donating cations enable efficient and stable acidic CO_2_ reduction in membrane electrode assemblies

**DOI:** 10.1093/nsr/nwaf312

**Published:** 2025-08-04

**Authors:** Shijia Feng, Ziang Liu, Dongfang Cheng, Yunfeng Hu, Sizhe Chen, Xinyuan Zhang, Jiabao Li, Xiaorui Dong, Tianyu Wang, Ziwei Wang, Yulun Wu, Ya Yin, Hongzhi Zheng, Philippe Sautet, Xiaojun Wang, Jia Zhu

**Affiliations:** National Laboratory of Solid State Microstructures, School of Sustainable Energy and Resources, Jiangsu Key Laboratory of Artificial Functional Materials, Collaborative Innovation Center of Advanced Microstructures, Frontiers Science Center for Critical Earth Material Cycling, Nanjing University, Nanjing 210008, China; College of Engineering and Applied Sciences, Nanjing University, Nanjing 210093, China; College of Engineering and Applied Sciences, Nanjing University, Nanjing 210093, China; Department of Chemical and Biomolecular Engineering, University of California, Los Angeles, Los Angeles, CA 90095, USA; College of Engineering and Applied Sciences, Nanjing University, Nanjing 210093, China; College of Engineering and Applied Sciences, Nanjing University, Nanjing 210093, China; National Laboratory of Solid State Microstructures, School of Sustainable Energy and Resources, Jiangsu Key Laboratory of Artificial Functional Materials, Collaborative Innovation Center of Advanced Microstructures, Frontiers Science Center for Critical Earth Material Cycling, Nanjing University, Nanjing 210008, China; School of Mechanical Engineering, Guangxi University, Nanning 530004, China; College of Engineering and Applied Sciences, Nanjing University, Nanjing 210093, China; National Laboratory of Solid State Microstructures, School of Sustainable Energy and Resources, Jiangsu Key Laboratory of Artificial Functional Materials, Collaborative Innovation Center of Advanced Microstructures, Frontiers Science Center for Critical Earth Material Cycling, Nanjing University, Nanjing 210008, China; College of Engineering and Applied Sciences, Nanjing University, Nanjing 210093, China; Key Laboratory of Mesoscopic Chemistry of Ministry of Education, School of Chemistry and Chemical Engineering, Nanjing University, Nanjing 210023, China; Key Laboratory of Mesoscopic Chemistry of Ministry of Education, School of Chemistry and Chemical Engineering, Nanjing University, Nanjing 210023, China; National Laboratory of Solid State Microstructures, School of Sustainable Energy and Resources, Jiangsu Key Laboratory of Artificial Functional Materials, Collaborative Innovation Center of Advanced Microstructures, Frontiers Science Center for Critical Earth Material Cycling, Nanjing University, Nanjing 210008, China; College of Engineering and Applied Sciences, Nanjing University, Nanjing 210093, China; Department of Chemical and Biomolecular Engineering, University of California, Los Angeles, Los Angeles, CA 90095, USA; Department of Chemistry and Biochemistry, University of California, Los Angeles, Los Angeles, CA 90095, USA; National Laboratory of Solid State Microstructures, School of Sustainable Energy and Resources, Jiangsu Key Laboratory of Artificial Functional Materials, Collaborative Innovation Center of Advanced Microstructures, Frontiers Science Center for Critical Earth Material Cycling, Nanjing University, Nanjing 210008, China; National Laboratory of Solid State Microstructures, School of Sustainable Energy and Resources, Jiangsu Key Laboratory of Artificial Functional Materials, Collaborative Innovation Center of Advanced Microstructures, Frontiers Science Center for Critical Earth Material Cycling, Nanjing University, Nanjing 210008, China; College of Engineering and Applied Sciences, Nanjing University, Nanjing 210093, China

**Keywords:** acidic CO_2_ reduction, cationic effects, proton-donating effects, NH_3_/NH_4_^+^ recirculation, membrane electrode assemblies

## Abstract

Electrochemical CO_2_ reduction (CO_2_R) in acidic membrane electrode assemblies (MEAs) represents a promising pathway for sustainable chemical production, but achieving high selectivity, low cell voltage and long-term stability remains challenging. Current approaches using alkali cations can promote selectivity through cationic effects, but relying on H_2_O as a weak proton donor results in high overpotential and severe precipitation, causing elevated cell voltage and poor operational stability. Here, we introduce NH_4_^+^ as a proton-donating cation that simultaneously addresses these challenges in acidic MEAs. As a cation, it electromigrates to the catalyst surface, stabilizing *CO_2_ intermediates and reducing localized H^+^ concentration for high selectivity. As a proton donor, it provides superior proton-donating ability compared to H_2_O when H^+^ mass transport is limited, which decreases the protonation barrier and reduces CO_2_R overpotential on CoPc@CNT, resulting in a lower cell voltage. Furthermore, NH_4_^+^ effectively donates protons to bicarbonate, promoting its decomposition at significantly lower temperatures compared to KHCO_3_, thereby enabling easy removal of precipitates through mild heating and maintaining an NH_3_/NH_4_^+^ recirculation system for operational stability. As a result, this approach achieves an average CO_2_-to-CO selectivity of 86% in acidic MEAs at 100 mA cm^−2^ and 60°C using CoPc@CNT–NH_2_ catalyst, with stable performance over 110 h at an average cell voltage of 2.84 V, corresponding to a 40.6% energy efficiency. This strategy advances acidic MEA-based CO_2_R toward practical implementation by simultaneously achieving high selectivity, low overpotential and stable operation.

## Teaser

Beyond enhancing selectivity via cation effects, this work employs the proton-donating ability of NH4^+^ to lower both the protonation barrier of CO_2_ reduction and the bicarbonate decomposition barrier, achieving high energy efficiency and stability in acidic membrane electrode assemblies.

## INTRODUCTION

Electrocatalytic CO_2_ reduction (CO_2_R) powered by renewable energy represents a promising strategy for converting CO_2_ into valuable chemical products, offering a sustainable pathway toward carbon-neutral chemical industry [1–3]. While both alkaline and acidic electrolyzers have been extensively studied for CO_2_ conversion [4,5], acidic electrolyzers offer unique advantages by preventing CO_2_ carbonation and eliminating CO_2_ crossover [6–8], thereby enabling higher single-pass CO_2_ conversion efficiency and avoiding the purification costs associated with CO_2_ and O_2_ mixing [9–11]. However, the implementation of acidic CO_2_R systems faces critical challenges in achieving both high energy efficiency and long-term stability. High energy efficiency requires the maintenance of high product selectivity while operating at low cell voltage. Addressing these challenges is crucial for realizing the full potential of acidic electrolyzers in industrial-scale sustainable chemical production.

The industrial implementation of CO_2_R requires electrolyzers that can deliver both high energy efficiency and scalability [12]. Among various configurations, membrane electrode assemblies (MEAs) represent the most promising pathway toward commercialization [13]. By eliminating liquid catholyte and enabling zero-gap operation, MEAs significantly reduce ohmic losses and system complexity, providing a practical route to industrial-scale production. While both cation exchange membranes (CEMs) and anion exchange membranes can be utilized in MEA systems [14,15], CEMs offer a distinctive acid-environment advantage in preventing CO_2_ carbonation and crossover [16]. However, this acidic environment poses a significant challenge for CO_2_R selectivity, with a limited selectivity of 37% in pure acidic MEAs [17].

To address the selectivity challenge in acidic MEAs, two main strategies have been developed to modify the local reaction environment. The first strategy employs alkali cations to enhance product selectivity by stabilizing reaction intermediates and reducing local H^+^ concentration [6,9,11,18,19]. However, the fundamental challenge of bicarbonate precipitation emerges during extended operation [20,21], affecting CO_2_ transport and electrolyte stability, which limits continuous operation to around 20 h [10,22]. The second strategy utilizes polymer cations to circumvent precipitation issues [16,23–25]; however, the limited density of fixed cations poses an intrinsic ionic conductivity challenge in acidic MEAs. To suppress the hydrogen evolution reaction (HER) and achieve high CO_2_R selectivity, this strategy requires operation in weak acid or pure water environments, where the significant ohmic losses result in prohibitively high cell voltages (3.6–4.0 V) and low energy efficiency (22%–26%) even at moderate current densities (100 mA cm^−2^) [16,25], presenting a major barrier to practical implementation.

The progress achieved through alkali cations and polymer cations demonstrates that the cationic effects, including stabilizing reaction intermediates and regulating local pH, are essential for high CO_2_R selectivity. To make acidic CO_2_ electrolysis practically viable, we need to maintain this high selectivity while addressing the remaining challenges in cell voltage and stability. The decomposition of bicarbonate requires proton participation, and a more efficient proton source could facilitate bicarbonate decomposition, preventing precipitation and enhancing operational stability. Moreover, our previous mechanistic studies on CoPc@CNT catalyst reveal that protonation is the potential-determining step in the CO_2_R process [17]. The reliance on water as a proton donor, with its inherently weak proton-donating ability (i.e. a high thermodynamic barrier for proton donation), contributes to a high overpotential. A stronger proton donor could potentially lower the barrier, thereby reducing cell voltage and improving energy efficiency. Therefore, designing a system that can provide efficient ability of proton donating while maintaining the beneficial effects of cationic species becomes crucial for achieving high selectivity, low cell voltage and long-term stability in acidic CO_2_ electrolysis.

**Figure 1.**
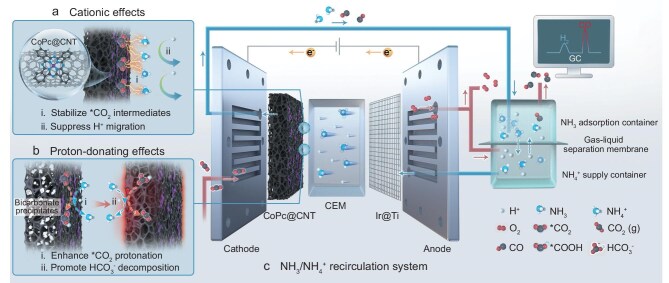
Schematic of NH_4_^+^-mediated CO_2_R in acidic MEAs. NH_4_^+^ enables efficient and stable CO_2_R with cationic, proton-donating effects, supported by an NH_3_/NH_4_^+^ recirculation system. (a) Cationic effects: NH_4_^+^ migrates and accumulates at the electrode surface, stabilizing *CO_2_ intermediates through dipole–electric field interactions, and suppressing H^+^ migration due to the competitive migration of NH_4_^+^. (b) Proton-donating effects: NH_4_^+^ donates protons more readily than water, facilitating the protonation of *CO_2_ and lowering the CO_2_R overpotential. Furthermore, NH_4_^+^ effectively donates protons to bicarbonate, promoting bicarbonate decomposition. (c) NH_3_/NH_4_^+^ recirculation system: NH_4_HCO_3_ precipitates decompose at low temperatures into NH_3_, which is protonated by H^+^ in an ‘NH_3_ adsorption container’ to form NH_4_^+^. These NH_4_^+^ ions diffuse back to an ‘NH_4_^+^ supply container’, ensuring a steady NH_4_^+^ supply.

To address these challenges, we propose that a proton-donating cation could provide an ideal solution by combining the beneficial properties of cations with enhanced proton-donating ability (Fig. 1). Our computational and experimental studies identify NH_4_^+^ as an optimal candidate that simultaneously fulfills the dual requirements of our design strategy. First, like alkali cations and polymer cations, NH_4_^+^ maintains high CO_2_-to-CO selectivity by stabilizing reaction intermediates and reducing local H^+^ concentration (Fig. 1a). Second, and uniquely, NH_4_^+^ acts as a superior proton donor compared to water under mass transport-limited conditions for H^+^ (Fig. 1b), effectively lowering the protonation barrier and reducing the CO_2_R overpotential on CoPc@CNT by 280 mV at 100 mA cm^−2^. This reduction in cathodic overpotential contributes to lowering of the cell voltage of the MEA system, thereby enhancing energy efficiency. Furthermore, NH_4_^+^ proves to be a more efficient proton donor than HCO_3_^−^, leading to a significantly lower decomposition temperature for NH_4_HCO_3_ relative to KHCO_3_ (Fig. 1b). This property facilitates the easy removal of NH_4_HCO_3_ precipitates via mild heating, allowing NH_4_^+^ to function within an NH_3_/NH_4_^+^ recirculation system (Fig. 1c). Consequently, the facile thermal removal of precipitated salts enables continuous NH_4_^+^ supply, fundamentally resolving the long-standing salt precipitation issue in acidic MEAs and maintaining consistent performance throughout the reaction. As a result, CoPc@CNT–NH_2_ catalyst achieves an average CO_2_-to-CO selectivity of 86% at a current density of 100 mA cm^−2^ and 60°C in acidic MEAs, with stable performance sustained over 110 h with an average cell voltage of 2.84 V, corresponding to an energy efficiency of 40.6%. To the best of our knowledge, this represents the most stable system among acidic MEAs while maintaining high energy efficiency throughout the CO_2_R operation. With salt precipitation issues resolved, the system's long-term stability now depends primarily on catalyst stability, suggesting that more stable catalysts would further enhance operational longevity. These findings present a novel strategy for overcoming the inherent challenges of acidic CO_2_R, offering a sustainable pathway toward carbon-neutral chemical production.

## RESULTS AND DISCUSSION

### The cationic effects of NH_4_^+^ on acidic CO_2_R

Unlike conventional alkali cations, NH_4_^+^ possesses dual functionality as both a cation and a proton donor, making it uniquely suited for acidic CO_2_R. To understand how NH_4_^+^ enhances CO_2_R selectivity through its cationic properties, we first investigate its effects on the reaction interface using computational calculations.

**Figure 2.**
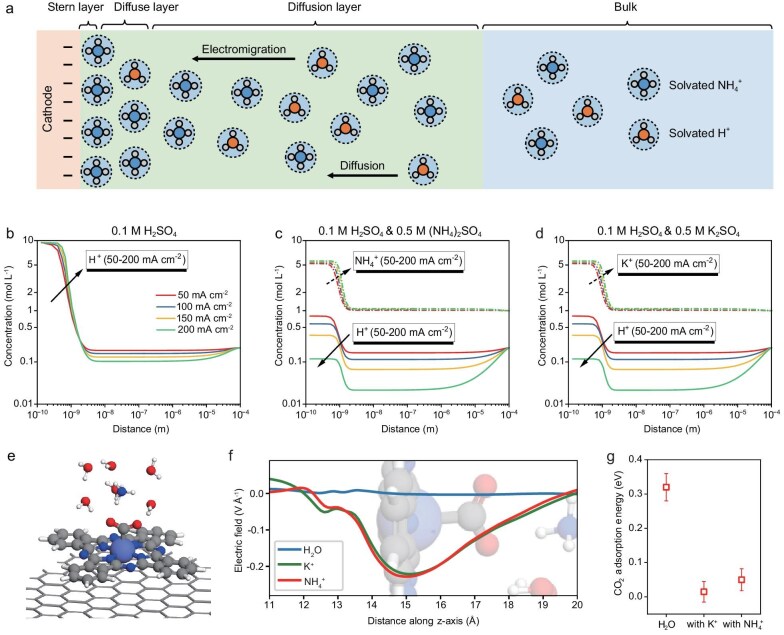
The cationic effects of NH_4_^+^ on acidic CO_2_R. (a) Schematic of ion transport near the cathode based on the GMPNP model, showing the Stern layer, diffuse layer and diffusion layer, with electromigration and diffusion processes for solvated ions (H^+^, NH_4_^+^ and K^+^). (b–d) Ion concentration profiles as a function of distance from the cathode for different electrolytes: (b) 0.1 M H_2_SO_4_, (c) 0.1 M H_2_SO_4_ and 0.5 M (NH_4_)_2_SO_4_, and (d) 0.1 M H_2_SO_4_ and 0.5 M K_2_SO_4_, simulated under varying current densities. (e) Atomic structure of NH_4_^+^-containing electrolyte with CO_2_ adsorption on CoPc@CNT. (f) Averaged electric field distribution along the *z*-axis near the catalyst surface under different ion conditions (H_2_O, K^+^ and NH_4_^+^). (g) Adsorption energy of CO_2_ on CoPc@CNT in the presence of H_2_O, K^+^ and NH_4_^+^.

COMSOL simulations are conducted to assess the effects of NH_4_^+^ on localized H^+^ concentration under reaction conditions. Given that cations significantly accumulate at the electrode surface under the influence of electric field, both spatial constraints and ion size must be considered in the simulations. Therefore, we employ the generalized modified Poisson-Nernst-Planck (GMPNP) model [20,26–28], which accounts for electric field effects, diffusion and reactions to precisely calculate species concentration distributions in the reaction region (Fig. 2a). The simulation details are provided in the Methods and Table S1 of the Supplementary data.

Both the CO_2_-to-CO conversion and HER similarly affect the electrode interface, as they involve proton consumption at the catalyst surface. The corresponding electrode reactions are as follows:


(1) \begin{eqnarray*}
{\mathrm{C}}{{\mathrm{O}}}_2 + 2{\mathrm{H}}_{{\mathrm{active}}}^ + + 2{{\mathrm{e}}}^ - = {\mathrm{CO}} + {{\mathrm{H}}}_2{\mathrm{O}},
\end{eqnarray*}

(2) \begin{eqnarray*}
2{\mathrm{H}}_{{\mathrm{active}}}^ + + 2{{\mathrm{e}}}^ - = {{\mathrm{H}}}_2.
\end{eqnarray*}

Here, ${\mathrm{H}}_{{\mathrm{active}}}^ + $ refers to the active protons involved in the HER or CO_2_R processes (Fig. S1). In the case of a pure acidic system, the sources of ${\mathrm{H}}_{{\mathrm{active}}}^ + $ are H^+^ and H_2_O. For the NH_4_^+^ system, ${\mathrm{H}}_{{\mathrm{active}}}^ + $ originates from H^+^, NH_4_^+^ and H_2_O. It is important to note that NH_4_^+^ and water supply ${\mathrm{H}}_{{\mathrm{active}}}^ + $ for HER or CO_2_R, and the resulting NH_3_ and OH^−^ quickly react with H^+^ when H^+^ is sufficient (Fig. S2), converting back to NH_4_^+^ and H_2_O:


(3) \begin{eqnarray*}
{\mathrm{NH}}_4^ + = {\mathrm{N}}{{\mathrm{H}}}_3 + {\mathrm{H}}_{{\mathrm{active}}}^ + ,
\end{eqnarray*}

(4) \begin{eqnarray*}
{\mathrm{N}}{{\mathrm{H}}}_3 + {{\mathrm{H}}}^ + = {\mathrm{NH}}_4^ + \left( {{\mathrm{fast}}} \right),
\end{eqnarray*}

(5) \begin{eqnarray*}
{{\mathrm{H}}}_2{\mathrm{O}} = {\mathrm{O}}{{\mathrm{H}}}^ - + {\mathrm{H}}_{{\mathrm{active}}}^ + ,
\end{eqnarray*}

(6) \begin{eqnarray*}
{\mathrm{O}}{{\mathrm{H}}}^ - + {{\mathrm{H}}}^ + = {{\mathrm{H}}}_2{\mathrm{O}}\left( {{\mathrm{fast}}} \right).
\end{eqnarray*}

From an overall reaction perspective, the protons consumed in the electrode reactions all originate from H^+^. These reactions establish the framework for analyzing species distribution during CO_2_R. We compare the species distributions in 0.1 M H_2_SO_4_ alone and with the addition of 0.5 M (NH_4_)_2_SO_4_, at current densities ranging from 50 to 200 mA cm^−2^ (Fig. 2b and c). In the pure acidic system (0.1 M H_2_SO_4_), a significant accumulation of H^+^ at the surface occurs under the influence of the electric field, approaching a saturation concentration of 9.50 mol L^−1^ (Fig. 2b). In the NH_4_^+^-containing system, under the influence of the electric field, NH_4_^+^ competes with H^+^ for accumulation at the surface. As the current density increases, the concentration of H^+^ gradually decreases, while the concentration of NH_4_^+^ continues to rise. At the current density of 100 mA cm^−2^, the surface H^+^ concentration in the NH_4_^+^-containing system is 0.58 M, 6% of that in the pure acidic system. At 200 mA cm^−2^, it even decreases to 0.12 M, 1.3% of the pure acidic system's concentration.

When comparing the effects of different cations, we found that NH_4_^+^ produces species distributions similar to those observed with K^+^ (Fig. 2d). This can be attributed to two main factors. First, both NH_4_^+^ and K^+^ are cations that compete with H^+^ for electromigration, which effectively reduces the localized concentration of H^+^. Second, the comparable hydrated radii and migration rates of NH_4_^+^ and K^+^ result in similar electromigration behaviors. As shown above, NH_4_^+^ effectively reduces the localized H^+^ concentration, thereby inhibiting the conversion of H^+^ to H_2_. These findings demonstrate that NH_4_^+^ effectively modulates the reaction interface by reducing local H^+^ concentration, which is crucial for suppressing the competing HER.

Beyond controlling H^+^ concentration, the accumulated NH_4_^+^ at the surface may create a localized electric field that stabilizes key reaction intermediates, particularly those with large dipole moments such as *CO_2_ [18]. To investigate this effect, we perform density functional theory (DFT) calculations using CoPc@CNT as a model catalyst system. This well-defined single-atom catalyst not only enables accurate computational modeling [29] but also demonstrates high activity under acidic conditions in our previous study [17], making it ideal for studying the role of NH_4_^+^ in acidic CO_2_R. Based on this, we examine the electric field distribution at the interface under various electrolyte conditions (Fig. 2e and f, and Fig. S3). Pure water without cations is used as the blank control, while K^+^, a well-known cation capable of creating a localized electric field [30,31], serves as the reference. In the presence of pure water, the interfacial electric field remains negligible due to the lack of significant charge separation between water molecules and the CoPc@CNT catalyst. However, upon introducing NH_4_^+^ into the electrolyte, a pronounced electric field develops at the interface, with the field strength peaking at *z* = 15 Å, corresponding to the position of adsorbed *CO_2_. It is important to note that the electric field strength shown here is *z*-averaged, and the local cation-induced electric field is significantly stronger in the vicinity of the cation. Thus, we anticipate a much stronger field strength near the active Co site when the cation is close to the Co. Interestingly, we found that the electric field induced by NH_4_^+^ closely resembles that generated by K^+^, suggesting that NH_4_^+^ and K^+^ can induce similar field–dipole interactions. As shown in Fig. 2g, both K^+^ and NH_4_^+^ significantly stabilize the *CO_2_ intermediate, increasing its binding energy by approximately 0.3 eV. This stabilization is attributed to the large dipole moment of *CO_2_, which is highly sensitive to the local electric field. The negative field stabilizes the dipole, thus favoring the activation of CO_2_. Since *H has a minimal dipole moment and is therefore less sensitive to the electric field, our previous work indicates that *H and *CO_2_ compete with each other in the absence of cations [17]. Consequently, the presence of cations is expected to have a more pronounced promotional effect on CO_2_R. Integrating the results from COMSOL simulations and DFT calculations, NH_4_^+^ electromigrates and accumulates at the catalyst surface, leading to a reduction in localized H^+^ concentration and stabilization of *CO_2_ intermediates, which ensures the selectivity for CO_2_-to-CO conversion.

Collectively, our computational calculations reveal that NH_4_^+^ serves two essential functions as a cation: reducing local H^+^ concentration through competitive electromigration, and stabilizing reaction intermediates via field–dipole interactions. These effects work synergistically to promote CO_2_R selectivity while suppressing the competing HER, addressing a key challenge in acidic CO_2_R systems.

### The proton-donating effects of NH_4_^+^ on acidic CO_2_R

Having established the role of NH_4_^+^ as a cation in modulating the reaction interface, we next investigate its function as a proton donor. While CO_2_R involves protonation as a key elementary step, the conventional proton source H_2_O (pKa = 14) presents a high kinetic barrier [32]. NH_4_^+^, with a significantly lower pKa of 9.25, could potentially serve as a more efficient proton donor. For our model catalyst CoPc@CNT, where protonation is the potential-determining step [17], this property of NH_4_^+^ may substantially reduce the overpotential required for CO_2_R.

**Figure 3.**
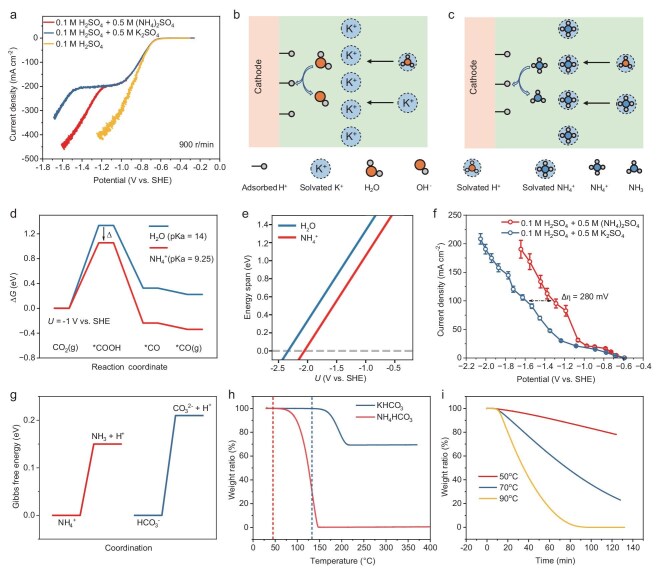
The proton-donating effects of NH_4_^+^ on acidic CO_2_R. (a) LSV curves for 0.1 M H_2_SO_4_ with 0.5 M (NH_4_)_2_SO_4_ or 0.5 M K_2_SO_4_, and for 0.1 M H_2_SO_4_ alone, measured using a rotating disk electrode at 900 r/min in an Ar-saturated environment. (b and c) Schematic illustration of ion distribution at the cathode in the presence of (b) K^+^, where H_2_O acts as the proton donor, and (c) NH_4_^+^, where NH_4_^+^ serves as the proton donor. Both K^+^ and NH_4_^+^ hinder the migration of H^+^ to the electrode surface. (d) Free energy diagrams for CO_2_R pathways on CoPc@CNT at −1 V vs SHE, comparing H_2_O and NH_4_^+^ as proton donors. (e) Calculated energy span as a function of potential for H_2_O and NH_4_^+^. (f) CO partial current density as a function of potential in 0.1 M H_2_SO_4_ electrolytes containing NH_4_^+^ or K^+^. (g) Gibbs free energy diagrams comparing the proton-donating abilities of NH_4_^+^ and HCO_3_^−^. (h) Thermogravimetric analysis of NH_4_HCO_3_ and KHCO_3_, showing the decomposition temperatures of bicarbonate species. (i) Weight loss profiles of NH_4_HCO_3_ at different temperatures.

To systematically investigate the proton-donating abilities of NH_4_^+^, we first employ rotating disk electrode (RDE) measurements to compare different proton sources under conditions where the mass transfer of H^+^ is limiting. Linear sweep voltammetry (LSV) measurements during HER are used to analyze the onset potentials at which NH_4_^+^ and H_2_O act as proton sources. Figure 3a shows the LSV results for HER at a rotation speed of 900 r/min in various acidic electrolytes. For the 0.1 M H_2_SO_4_ electrolyte, the HER current density increases as the potential becomes more negative, and the LSV curve follows the typical e-exponential behavior described by the Butler–Volmer equation (Fig. S4a) [33]. In contrast, for the electrolyte system with the addition of NH_4_^+^ and K^+^, the LSV curve exhibits distinct differences, with the curve clearly divided into three regions. For the NH_4_^+^-containing electrolyte, in the first region (−0.6 to −1.0 V), H^+^ reduction occurs, but the current density is significantly lower than in the pure acidic system (0.1 M H_2_SO_4_). This reduction is attributed to NH_4_^+^ lowering the localized H^+^ concentration, which leads to an elevated local pH, as confirmed by the RDE measurements (Fig. S5). In the second region (−1.0 to −1.2 V), the current density remains nearly constant despite increasingly negative potentials, which is characteristic of mass transport limitation [20]. In this case, the reactant is H^+^. This phenomenon is not observed under pure acidic conditions (Fig. S4a), indicating that the mass transport limitation of H^+^ is caused by cations. Higher plateau current densities observed with increased rotation speeds from 900 to 2500 r/min confirm that this potential range is H^+^ diffusion-limited (Fig. S4b). In the third region (potentials more negative than −1.2 V), the increased current density indicates the involvement of a new proton source in HER. Similarly, LSV curves obtained in an acidic solution containing K^+^ also display three distinct regions (Fig. 3a and Fig. S4c). The key difference lies in the onset potential of the third region, which corresponds to the potential at which the new proton source begins to participate in HER. In the K^+^-containing system, water acts as the proton source at −1.5 V (Fig. 3b), whereas in the NH_4_^+^ system, HER begins at −1.2 V. The different onset potentials confirm that NH_4_^+^, rather than water, serves as the proton source in the NH_4_^+^ system (Fig. 3c). The more positive onset potential in the NH_4_^+^ system demonstrates that NH_4_^+^ is a more effective proton donor than H_2_O. This characteristic is expected to lower the protonation barrier in the CO_2_R process, thereby reducing the overpotential and facilitating CO_2_R.

These experimental results clearly demonstrate the superior proton-donating ability of NH_4_^+^ compared to H_2_O. To understand the thermodynamic basis for this enhancement, we turn to DFT calculations. Our recent work demonstrates that the pH level in proton-coupled electron transfer (PCET) processes can be correlated with the pKa of the proton source [34]. The chemical potential of a coupled proton and electron is determined by the equilibrium with H_2_ gas at the hydrogen electrode as shown:


(7) \begin{eqnarray*}
{G}_{{{\mathrm{H}}}^ + /{e}^ - } = \frac{1}{2}{G}_{{{\mathrm{H}}}_2} - eU - \ln( {10} ){k}_{\mathrm{B}}T{\mathrm{p}}{{\mathrm{K}}}_{\mathrm{a}},
\end{eqnarray*}

where ${G}_{{{\mathrm{H}}}^ + /{e}^ - }$ is the Gibbs free energy of a coupled proton–electron pair, ${G}_{{{\mathrm{H}}}_2}$ is the Gibbs free energy of H_2_ in the gas phase at standard state, *U* is the electrode potential vs standard hydrogen electrode (SHE), ${k}_{\mathrm{B}}$ is the Boltzmann constant, *T* is the absolute temperature, and pK_a_ is the negative log_10_ of the acid dissociation constant of the relevant acid.

As protons are always reactants in these systems, lowering the pKa of the proton source decreases the overall reaction energy, thereby affecting both the equilibrium and onset potentials. When the H^+^ mass transfer is limited, water (with a pKa of 14) typically acts as the proton source. However, NH_4_^+^ (with a lower pKa of 9.25) can facilitate PCET processes more efficiently. The CO_2_ conversion to CO on CoPc@CNT proceeds through two key PCET steps: CO_2_(g) to *COOH, followed by *COOH to CO(g). As shown in Fig. 3d, the first step (CO_2_ to *COOH) is the potential-determining step, with *COOH being the intermediate with the highest energy level along the reaction pathway. When NH_4_^+^ is used as the proton source, there is a significant reduction in the overall energy span (Δ), as follows:


(8) \begin{eqnarray*}
\Delta \ = \ln ( {10} ){k}_{\mathrm{B}}T[{\mathrm{p}}{{\mathrm{K}}}_{\mathrm{a}}( {{{\mathrm{H}}}_2{\mathrm{O}}}) - {\mathrm{p}}{{\mathrm{K}}}_{\mathrm{a}}( {{\mathrm{NH}}_4^ + } ),
\end{eqnarray*}

where ${\mathrm{p}}{{\mathrm{K}}}_{\mathrm{a}}( {{{\mathrm{H}}}_2{\mathrm{O}}} )$ and ${\mathrm{p}}{{\mathrm{K}}}_{\mathrm{a}}( {{\mathrm{NH}}_4^ + } )$ are pKa values of H_2_O and NH_4_^+^. This is independent of the electrode potential, as depicted in Fig. 3e. Given that the CO_2_-to-CO reaction involves two PCET steps, the equilibrium potential when NH_4_^+^ is used as the proton source is reduced by 2Δ, approximately 0.56 V (Fig. S6). Thus, the pKa of the proton source significantly impacts both the energy span and equilibrium potential for CO_2_R to CO. A lower pKa results in a reduced energy span, implying slower kinetics according to the Bell–Evans–Polanyi (BEP) relationship when compared to that when water is used as the proton source. Meanwhile, the decrease in equilibrium potential provides a stronger thermodynamic driving force for CO_2_ conversion to CO when NH_4_^+^ is present. Therefore, compared to water, NH_4_^+^ offers superior kinetic and thermodynamic advantages, which can effectively reduce the overpotential in CO_2_R.

To confirm that NH_4_^+^ more effectively promotes the protonation process in CO_2_R compared to H_2_O, CO_2_R activity tests are operated by using a three-electrode flow cell. Unlike two-electrode MEAs, the three-electrode system can accurately evaluate the effect of cations on the cathode potential, minimizing interference from anode-related factors such as gas bubbles and device assembly during testing. Figure 3f and Fig. S7 shows that NH_4_^+^-mediated CO_2_R operates at a more positive reaction potential than K^+^-mediated CO_2_R, resulting in a lower overpotential. At a partial current density of 100 mA cm^−2^ for CO production, the overpotential with NH_4_^+^ is up to 280 mV lower than that with K^+^. In addition, as shown in Fig. S8, the overpotentials for CO_2_R mediated by Li^+^ and Na^+^ are also higher than that of NH_4_^+^. These confirm that NH_4_^+^, with its superior proton-donating ability compared to H_2_O, more effectively facilitates the protonation process on CoPc@CNT, thereby significantly reducing the overpotential.

The strong proton-donating ability of NH_4_^+^ plays dual beneficial roles in acidic CO_2_R. First, as demonstrated above, it effectively reduces the reaction overpotential. Second, this same property proves advantageous in addressing another critical challenge in CO_2_R systems—the formation and removal of bicarbonate precipitates. In both NH_4_^+^ and conventional cation systems, the reaction of CO_2_ with alkaline species (OH^−^ and NH_3_) leads to bicarbonate precipitation (KHCO_3_ and NH_4_HCO_3_), which can obstruct CO_2_ transport channels and compromise system stability. The key to removing these precipitates lies in their decomposition mechanism, which fundamentally involves proton transfer processes. Here, the superior proton-donating ability of NH_4_^+^ plays a crucial role in facilitating this decomposition. Given that thermal treatment can decompose bicarbonate precipitates, understanding the decomposition mechanisms of KHCO_3_ and NH_4_HCO_3_ is crucial for developing effective mitigation strategies. The decomposition of bicarbonates requires proton transfer processes, as the formation of final products (CO_2_ and H_2_O) necessitates the combination of protons with bicarbonate ions. The key difference between KHCO_3_ and NH_4_HCO_3_ lies in their proton transfer pathways during decomposition. The decomposition of KHCO_3_ requires intermolecular proton transfer between adjacent molecules, while NH_4_HCO_3_ can decompose through intramolecular proton transfer:


(9) \begin{eqnarray*}
{\mathrm{HCO}}_3^ - \left( {\mathrm{a}} \right) = {{\mathrm{H}}}^ + \left( {\mathrm{a}} \right) + {\mathrm{CO}}_3^{2 - }\left( {\mathrm{a}} \right),
\end{eqnarray*}

(10) \begin{eqnarray*}
{{\mathrm{H}}}^ + \left( {\mathrm{a}} \right) + {\mathrm{HCO}}_3^ - \left( {\mathrm{b}} \right) = {{\mathrm{H}}}_2{\mathrm{O}} + {\mathrm{C}}{{\mathrm{O}}}_2,
\end{eqnarray*}

(11) \begin{eqnarray*}
{\mathrm{NH}}_4^ + = {\mathrm{N}}{{\mathrm{H}}}_3 + {{\mathrm{H}}}^ + ,
\end{eqnarray*}

(12) \begin{eqnarray*}
{{\mathrm{H}}}^ + + {\mathrm{HCO}}_3^ - = {{\mathrm{H}}}_2{\mathrm{O}} + {\mathrm{C}}{{\mathrm{O}}}_2.
\end{eqnarray*}

Here, a and b represent two adjacent KHCO_3_ molecules. The energy difference between these decomposition pathways primarily stems from the distinct proton donation barriers of HCO_3_^−^ and NH_4_^+^. In KHCO_3_, protons must transfer between neighboring molecules, creating a higher energy barrier. In contrast, NH_4_HCO_3_ contains its own proton source (NH_4_^+^), enabling more efficient intramolecular proton transfer.

DFT calculations indicate that the proton-donation barrier for NH_4_^+^ is lower than that for HCO_3_^−^ (Fig. 3g). This implies that the decomposition temperature of NH_4_HCO_3_ is substantially lower than that of KHCO_3_. Thermogravimetric (TG) analysis further corroborates this finding (Fig. 3h and Fig. S9), with decomposition temperatures of NH_4_HCO_3_ and KHCO_3_ observed at 45°C and 133°C, respectively. Moreover, the decomposition of NH_4_HCO_3_ does not yield solid residues, allowing for its complete decomposition. As the temperature increases, the decomposition rate of NH_4_HCO_3_ accelerates (Fig. 3i). In contrast, KHCO_3_ decomposes to form K_2_CO_3_, which is more thermally stable and resists further decomposition. This makes it challenging to fully remove KHCO_3_ through mild heating.

These findings comprehensively demonstrate the dual advantages of NH_4_^+^ as a proton donor in acidic CO_2_R. First, its lower pKa compared to H_2_O enables more efficient proton transfer, leading to a significant reduction in overpotential (up to 280 mV lower than K^+^-mediated systems) during CO_2_R. Second, while both NH_4_^+^ and K^+^ systems face challenges from bicarbonate precipitation, the intramolecular proton transfer ability of NH_4_HCO_3_ enables its complete decomposition at mild temperatures (45°C vs 133°C for KHCO_3_). These unique properties suggest that NH_4_^+^-mediated systems can achieve cell voltage through enhanced proton donation and good stability through facile precipitate removal, addressing two key challenges in acidic CO_2_R.

### NH_3_/NH_4_^+^ recirculation system enables stable CO_2_R in acidic MEA

Based on our understanding of the functions of NH_4_^+^ and the thermal decomposition behavior of NH_4_HCO_3_, we design an NH_3_/NH_4_^+^ recirculation system to address a critical challenge in MEA operation: maintaining stable NH_4_^+^ supply (Fig. 1c and Fig. S10). In MEA devices, NH_4_^+^ at the cathode interface is sourced from an ‘NH_4_^+^ supply container’, while bicarbonate precipitated at the cathode consumes and decreases its concentration in the supply container. Insufficient NH_4_^+^ at the cathode would weaken its crucial roles in stabilizing reaction intermediates, moderating local acidity and providing protons for CO_2_R. Our system incorporates a mild heating strategy to achieve dynamic equilibrium of NH_4_^+^ concentration. The anolyte contains both NH_4_^+^ and H^+^ ions, with NH_4_^+^ migrating to the cathode under the electric field to fulfill its multiple functions. When NH_4_HCO_3_ precipitation occurs, mild heating facilitates its decomposition into NH_3_, CO_2_ and H_2_O. The gaseous NH_3_ can be captured by an ‘NH_4_^+^ adsorption container’, where it reacts with H^+^ to regenerate NH_4_^+^. These regenerated NH_4_^+^ ions can then diffuse through the gas–liquid separation membrane to the ‘NH_4_^+^ supply container’. This NH_4_^+^/NH_3_ cycling system maintains sufficient NH_4_^+^ concentration at the cathode to sustain both its cationic and proton-donating effects. In contrast, K^+^-mediated MEAs lack such a self-regulating mechanism. Once K^+^ is lost to KHCO_3_ precipitation, it cannot be readily recovered through thermal decomposition and gas-phase transport, leading to continuous depletion of K^+^ supply and eventual system failure. Therefore, this NH_3_/NH_4_^+^ recirculation system provides a robust foundation for achieving stable CO_2_R in acidic MEAs.

**Figure 4.**
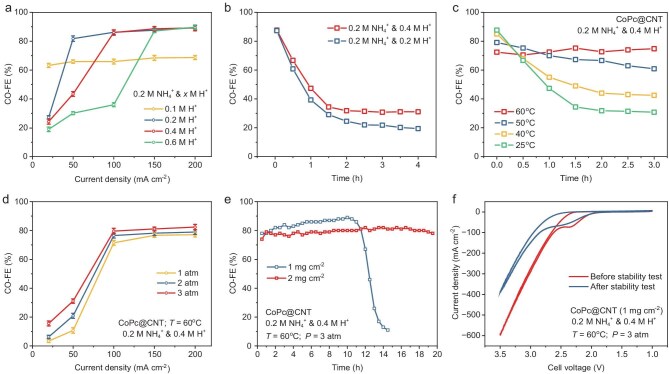
NH_3_/NH_4_^+^ recirculation system enables stable CO_2_R in acidic MEA. (a) CO-FE tests at various H^+^ concentrations with 0.2 M NH_4_^+^ in the anolyte on CoPc@CNT. (b) Stability tests with anolytes containing 0.2 M NH_4_^+^ and either 0.2 M or 0.4 M H^+^. (c) CO-FE tests under temperatures from 25 to 60°C, using 0.2 M NH_4_^+^ and 0.4 M H^+^ in the anolyte at 100 mA cm^−2^. (d) CO-FE tests under CO_2_ pressures of 1–3 atm at 60°C under identical conditions. (e) Stability tests at 100 mA cm^−2^, 3 atm and 60°C, comparing catalyst loadings of 1 and 2 mg cm^−2^. (f) Polarization curves recorded before and after stability testing at 1 mg cm^−2^ catalyst loading.

Before experimentally verifying the stability of NH_4_^+^-mediated acidic MEAs, anolyte composition should be first determined, especially for H^+^ concentration. The H^+^ concentration in the anolyte plays a critical role in both reaction selectivity and system stability through two mechanisms [10,22]. First, H^+^ depletion at the cathode interface is necessary for achieving high CO_2_R selectivity by suppressing the competing HER. Second, H^+^ helps neutralize the *in situ*-formed HCO_3_^−^ in the diffusion layer, preventing undesired salt precipitation. These dual roles of H^+^ create competing requirements for its concentration. At low H^+^ concentrations (e.g. 0.1 M), while H^+^ depletion is readily achieved at low current densities favoring CO_2_R selectivity, the insufficient H^+^ cannot effectively neutralize the HCO_3_^−^ formed from the reaction between NH_3_ (produced by NH_4_^+^ proton donation), CO_2_ and H_2_O, leading to salt precipitation. Conversely, higher H^+^ concentrations (e.g. 0.6 M) better prevent salt precipitation but require impractically high current densities (>150 mA cm^−2^) to achieve the H^+^ depletion necessary for high selectivity. To address this trade-off, we fixed the NH_4_^+^ concentration at 0.2 M while varying H^+^ concentrations. SO_4_^2−^ was chosen as the counter anion for its chemical inertness at both electrodes. Selectivity analysis (Fig. 4a and Fig. S11) reveals that intermediate H^+^ concentrations (0.2 and 0.4 M) achieve the best balance. While both concentrations show comparable CO selectivity at 100 mA cm^−2^, the higher H^+^ concentration (0.4 M) demonstrates better stability by more effectively preventing salt precipitation (Fig. 4b). Therefore, we determine the anolyte composition to be 0.1 M (NH_4_)_2_SO_4_ and 0.2 M H_2_SO_4_, which balances high selectivity with good stability at our target current density of 100 mA cm^−2^. Notably, the NH_4_^+^/H^+^ ratio used in the MEA system is significantly lower than that in flow cells, as the absence of flowing catholyte in MEAs minimizes the convective impact on the local pH.

The temperature is another key parameter for system performance, as it directly influences the NH_4_HCO_3_ decomposition. Figure 4c illustrates that as the temperature increases, stability improves gradually, with stability at 60°C remaining essentially unchanged over a period of 3 h. However, this enhancement in stability comes at the expense of CO selectivity, which decreases from 90% to 72%. This decrease is primarily attributed to the greater influence of temperature on the kinetics of the HER compared to CO_2_R, with elevated temperatures significantly enhancing HER kinetic activity. To enhance selectivity, increasing the CO_2_ partial pressure effectively reduces the CO_2_ adsorption barrier and improves the competitiveness of CO_2_R relative to HER. Considering that CO_2_ is typically stored and transported under pressurized conditions (50–100 atm), the use of CO_2_ at pressures below 10 atm does not require additional energy input. Figure 4d (details in Fig. S12) shows that increasing the pressure from 1 to 3 atm leads to an improvement in CO_2_R selectivity, increasing from 73% to 80% at a current density of 100 mA cm^−2^. However, further pressure increases are not pursued, as they have a minimal effect on selectivity while significantly increasing the demands on reactor gas tightness.

Under these conditions, we evaluate the stability of NH_4_^+^-mediated MEAs. The system demonstrates stable operation at 100 mA cm^−2^ for 10 h (Fig. 4e). Interestingly, we observe an increase in CO_2_R selectivity from 80% to 90% during the initial 10 h, which we attribute to gradual catalyst deactivation. As the number of active catalytic sites decreases, the increased current density per site further reduces local H^+^ concentration, suppressing HER and enhancing CO_2_R selectivity. This catalyst deactivation is confirmed by the decreased current density in post-stability cyclic voltammetry measurements (Fig. 4f). Notably, increasing the catalyst loading from 1 to 2 mg cm^−2^ extends the stable operation period from 10 to 20 h (Fig. 4e and Fig. S13). These results demonstrate that our NH_3_/NH_4_^+^ recirculation strategy effectively solves the salt precipitation challenge in acidic MEAs, with system stability now primarily limited by catalyst deactivation rather than precipitate-related issues. This finding motivates us to focus on catalyst engineering to further improve the stability of acidic CO_2_R systems.

### NH_4_^+^-mediated stable CO_2_R on CoPc@CNT–NH_2_

Having established an effective NH_3_/NH_4_^+^ recirculation strategy, we next focus on improving catalyst stability to achieve stable operation. The deactivation of CoPc@CNT primarily stems from Co atoms detaching from the phthalocyanine ring during the reaction [35]. Previous studies have shown that CoPc supported on amino-functionalized CNT (CNT–NH_2_) exhibits enhanced electrochemical stability [36], as the lone-pair electrons from –NH_2_ groups can coordinate axially with Co atoms, preventing their detachment. Based on this insight, we synthesize the molecularly dispersed catalyst CoPc@CNT–NH_2_ (Figs S14–S16), and evaluate its performance in acidic MEAs with a catalyst loading of 2 mg cm^−2^.

**Figure 5.**
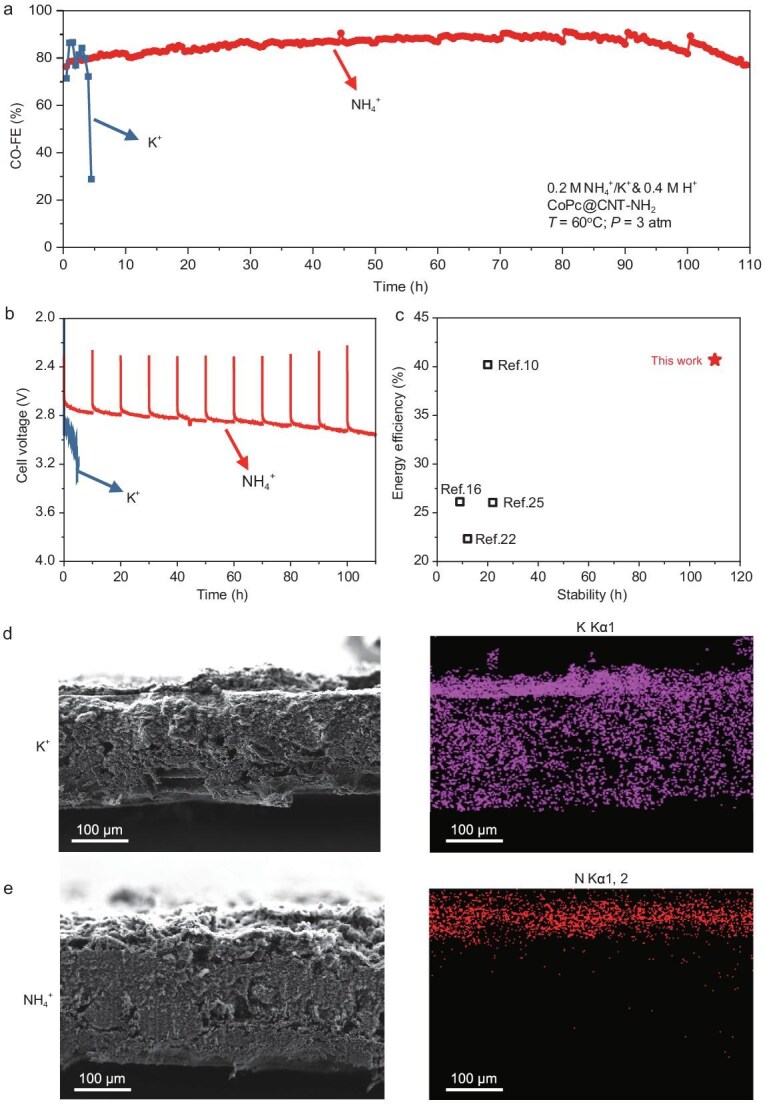
NH_4_^+^-mediated stable CO_2_R on CoPc@CNT–NH_2_. (a and b) Comparison of stability tests for NH_4_^+^- and K^+^-mediated CO_2_R systems at 0.2 M NH_4_^+^/K^+^ and 0.4 M H^+^, using CoPc@CNT–NH_2_ catalyst under conditions of 60°C and 3 atm CO_2_ at 100 mA cm^−2^. (c) Comparison of energy efficiency and stability with literature reports. (d) Cross-sectional SEM image and EDS mapping of gas diffusion electrode from the K^+^-mediated system, showing extensive salt precipitation throughout the electrode structure. (e) Cross-sectional SEM image and EDS mapping of gas diffusion electrode from the NH_4_^+^-mediated system, with N signals confined to the catalyst layer, confirming stable operation without salt precipitation.

To further mitigate the effects of salt precipitation on device performance during prolonged stability testing, we implement an operational protocol consisting of 10-hour reaction cycles followed by 1-hour intermittent pauses. These strategic interruptions serve dual purposes: first, they allow for the maintenance of optimal CO_2_ humidification conditions through the replenishment of water in the humidification bottle, otherwise dry CO_2_ would remove water from the cathode region, accelerating salt precipitation; second, they provide sufficient time for the complete decomposition of accumulated NH_4_HCO_3_, preventing its precipitation within the system. Based on this, the CoPc@CNT–NH_2_ catalyst maintains an average CO_2_-to-CO selectivity of 86% at 100 mA cm^−2^ for 110 h at an average cell voltage of 2.84 V, corresponding to a 40.6% energy efficiency (Fig. 5a and b, and Fig. S17). To the best of our knowledge, this represents the most stable system among acidic MEAs while maintaining high energy efficiency throughout the CO_2_R operation (Fig. 5c and Table S2). Furthermore, compared to the most stable neutral MEA systems reported recently [37] (Table S3), our system exhibits clear advantages in both single-pass conversion efficiency (Fig. S18) and cell voltage (Tables S3 and S4), which translate into significantly reduced energy consumption (Fig. S19) and operational costs (Fig. S20).

It should be noted that the system's stability is currently constrained by catalyst degradation rather than salt participation issues. Inductively coupled plasma analysis reveals a significant decrease in Co content in CoPc@CNT–NH_2_ after electrolysis (Fig. S21 and Table S5). This is further supported by transmission electron microscopy observations showing reduced Co signal intensity (Figs S15 and S22), and X-ray photoelectron spectroscopy results indicating a decline in the Co–N ratio (Fig. S23). Meanwhile, X-ray diffraction analysis shows no evidence of new phase formation (Fig. S24). Together, these characterizations confirm that the primary degradation pathway of CoPc@CNT–NH_2_ involves the leaching of Co from the phthalocyanine structure. Notably, the Co leaching rate in CoPc@CNT–NH_2_ is one-fifth of that in CoPc@CNT (Table S5), which correlates well with the slower rate of cell voltage increase observed during operation (Fig. S25). Moreover, the NH_4_^+^ concentration remains stable in both the NH_3_ adsorption container and the NH_4_^+^ supply container, and no NH_3_ leakage is detected (Fig. S26), ruling out the possibility of instability arising from the NH_3_/NH_4_^+^ recirculation system. These findings suggest that further development of more stable catalysts could directly lead to extended device lifetime.

To validate the effectiveness of our NH_4_^+^-mediated system, we conduct comparative tests with conventional K^+^-mediated MEAs under identical conditions (Fig. 5a). Due to the weaker proton-donating ability of H_2_O compared to NH_4_^+^, K^+^-mediated MEAs exhibit higher CO-Faradaic efficiency (CO-FE), which is consistent with results obtained in flow cell configurations (Fig. S7c). However, its weaker proton-donating ability also leads to a significantly higher cell voltage in the K^+^-mediated MEA compared to the NH_4_^+^-mediated MEAs (Fig. 5b). Moreover, the K^+^-mediated system shows significant CO-FE fluctuations within the first 4 h (Fig. 5b and Fig. S27), followed by rapid deactivation. This instability manifests through several observable phenomena: a notable decrease in product outlet flow rate, declining flowmeter readings (Fig. S28 and Table S6), and visible salt deposits blocking the gas channel outlet upon post-reaction inspection (Fig. S29). These observations indicate that salt precipitation severely compromises CO_2_ transport by blocking gas channels. Therefore, while the NH_4_^+^ system sacrifices a small degree of selectivity due to its stronger proton-donating ability, it significantly lowers the cell voltage and effectively eliminates the issue of salt precipitation, leading to dramatically improved operational stability. This trade-off is well justified for long-term electrolysis performance.

The contrasting stability of these systems is further evidenced by post-reaction scanning electron microscopy energy-dispersive spectroscopy (SEM-EDS) analysis of the gas diffusion electrodes. In K^+^-mediated MEAs (Fig. 5d), K^+^ is detected throughout the entire electrode structure, including the catalyst layer, microporous layer and fiber layer, confirming extensive salt precipitation. In contrast, NH_4_^+^-mediated MEAs (Fig. 5e) show nitrogen signals confined to the catalyst layer, likely originating from the catalyst components (CoPc structure and CNT–NH_2_) and residual nitrogen species. This localized distribution confirms the absence of salt precipitation in our NH_4_^+^-mediated system, validating its effectiveness in maintaining stable operation.

These comprehensive results demonstrate the successful development of a stable acidic CO_2_R MEAs through two key innovations: the NH_3_/NH_4_^+^ recirculation strategy that ensures the stable supply of NH_4_^+^, and the modified CoPc@CNT–NH_2_ catalyst that enables extended operation. The combination achieves stable CO_2_R performance with over 80% CO selectivity at 100 mA cm^−2^ for 110 h, representing a significant advancement in acidic MEA systems.

Compared with organic amines, NH_4_^+^ salts are among the most readily available and cost-effective proton-donating cations, making them an ideal and practical candidate for fundamental investigation. Therefore, NH_4_^+^ is chosen as the primary focus in this work. Meanwhile, common organic amines such as CH_3_NH_3_^+^ and C_2_H_5_NH_3_^+^ introduce longer carbon chains, which tend to decrease the solubility of bicarbonate salts and increase their decomposition temperature. These properties exacerbate salt precipitation issues and complicate the stability. Addressing such challenges would require precise molecular design and structural tuning, which lie well beyond the scope of the present study. Nevertheless, organic amines offer a unique advantage in their structural tunability. In future studies, we aim to exploit this tunability to improve bicarbonate solubility and lower decomposition temperatures, thereby enabling further reductions in operating temperature.

## CONCLUSION

In this work, we demonstrate an effective strategy to achieve both high energy efficiency and stability in acidic CO_2_R by utilizing NH_4_^+^ as a proton-donating cation. Our systematic investigations reveal two distinct roles of NH_4_^+^ in promoting CO_2_R performance. As a cation, NH_4_^+^ reduces surface H^+^ concentration and stabilizes *CO_2_ intermediates, leading to enhanced CO selectivity, as supported by DFT calculations and COMSOL simulations. As an efficient proton donor, NH_4_^+^ offers the advantages over both H_2_O and HCO_3_^−^: it reduces the CO_2_R overpotential on CoPc@CNT by 280 mV at 100 mA cm^−2^ through lowered protonation barriers, and enables facile removal of salt precipitation due to the significantly lower decomposition temperature of NH_4_HCO_3_ compared to KHCO_3_. Based on this low-temperature decomposition behavior, an NH_3_/NH_4_^+^ recirculation system is further established to ensure a stable supply of NH₄⁺ during electrolysis. As a result, the NH_4_^+^-mediated CoPc@CNT–NH_2_ system achieves an average CO selectivity of 86% at 100 mA cm^−2^ with stable performance for 110 h at an average cell voltage of 2.84 V, corresponding to a 40.6% energy efficiency. To the best of our knowledge, this represents the first acidic MEA system that simultaneously achieves high energy efficiency (40.6%) over 110 h, addressing a long-standing challenge in CO_2_R technology. Notably, the stability of the system is now determined by catalyst stability rather than salt precipitation, indicating that advances in catalyst robustness would extend system lifetime. Beyond the demonstrated high performance of the NH_4_^+^-mediated CoPc@CNT–NH_2_ system achieving stable CO production at acidic MEA, this strategy provides insights for designing next-generation acidic CO_2_R systems. The dual functionality principle established here could guide the development of other proton-donating species, particularly organic amines with tunable substituents, where the proton-donating ability can be systematically modulated through electronic and steric effects to improve CO_2_R performance.

## Supplementary Material

nwaf312_Supplemental_File
