# Supplementary material for: Proton-donating cations enable efficient and stable acidic CO2 reduction in membrane electrode assemblies
Source: Natl Sci Rev. 2025 Aug 4;12(10):nwaf312. doi: 10.1093/nsr/nwaf312 (PMC12462616; doi:10.1093/nsr/nwaf312)
Supplement: nwaf312_Supplemental_File [file nwaf312_supplemental_file.pdf]

Supporting Information

Proton-donating Cations Enable Efficient and Stable Acidic CO_2 Reduction in Membrane Electrode Assemblies

Shijia Feng[1,2,7], Ziang Liu[2,7], Dongfang Cheng[3,7], Sizhe Chen[2], Xinyuan Zhang[1], Jiabao Li[4], Xiaorui Dong[2], Tianyu Wang[1], Ziwei Wang[2], Yulun Wu[5], Ya Yin[5], Hongzhi Zheng[1,2], Philippe Sautet[3,6], Xiaojun Wang[1*], Jia Zhu[1,2*]

[1]National Laboratory of Solid State Microstructures, School of Sustainable Energy and Resources, Jiangsu Key Laboratory of Artificial Functional Materials, Collaborative Innovation Center of Advanced Microstructures, Frontiers Science Center for Critical Earth Material Cycling, Nanjing University, Jiangsu, P.R. China

[2]College of Engineering and Applied Sciences, Nanjing University, Nanjing 210093, P.R. China

[3]Department of Chemical and Biomolecular Engineering, University of California, Los Angeles, Los Angeles, California 90095, United States

[4]School of Mechanical Engineering, Guangxi University, Nanning 530004, P.R. China

[5]Key Laboratory of Mesoscopic Chemistry of Ministry of Education, School of Chemistry and Chemical Engineering, Nanjing University, Nanjing, 210023, P. R. China

[6]Department of Chemistry and Biochemistry, University of California, Los Angeles, Los Angeles, California 90095, United States

[7]These authors contributed equally to this work.

*Correspondence: jiazhu@nju.edu.cn, xiaojunwang@nju.edu.cn

Methods

Fabrication of catalysts. All chemicals were used without further purification.

The synthesis of CoPc@CNT is carried out following our previous work. 200 mg of multi-walled CNTs (Aladdin) are dispersed in 100 ml of CoPc (AR, Energy Chemical)-saturated DMF (AR, Sinopharm Chemical Reagent) solution using sonication for 2 hours to form a uniform suspension. The suspension is then stirred for an additional 7 days. Afterward, the mixture is centrifuged, and the resulting precipitate is washed sequentially with DMF, ethanol, and ultra-pure water. Finally, the precipitate is lyophilized to obtain the final product.

The synthesis method for CoPc@CNT-NH_2 (Aladdin) follows the same procedure as that for CoPc@CNT, with the only modification being the use of CNT-NH_2 instead of CNT.

Fabrication of gas diffusion electrode of CoPc@CNT (or CoPc@CNT-NH_2): Catalyst ink is prepared by dispersing 4 mg of CoPc@CNT (or CoPc@CNT-NH_2) in a mixture of 10 μL Nafion solution (DuPont, 5 ωt%) and 2 mL ethanol using sonication. Working electrodes are then prepared by drop-drying 2 mL of the catalyst ink onto hydrophobic carbon paper (Sigracet 28BC) to cover an area of 4 cm^2, achieving a catalyst loading of 1 mg cm^{-2}. To achieve a loading of 2 mg cm^{-2}, the amounts of catalyst, Nafion, and ethanol should be scaled up accordingly.

Material characterizations. The microscopic structures are characterized by SEM (Tescan, MIRAS3 FE-SEM). Energy dispersive X-ray spectroscopy is performed on Zeiss Ultra 55. Thermogravimetric tests are performed using a PerkinElmer TGA4000. Rotating disk electrode (RDE) measurements are carried out with a PINE AFMSRCE.

Electrochemical measurements in flow cells. All electrochemical measurements are conducted using a Corrtest multichannel workstation (CS310X). The experiments are performed in a custom-designed flow cell with three chambers (gas, catholyte, and anolyte), where a carbon paper-based gas-diffusion electrode is positioned between the gas and catholyte chambers, with an effective area of 1 × 1 cm^2. No special pre-treatment or rinsing process is required for the gas-diffusion electrodes before electrochemical tests. Prior to experimentation, the flow cell is disassembled and rinsed five times with ultra-pure water (18.2 MΩ·cm). A Nafion 115 cation-exchange membrane is used to separate the catholyte and anolyte chambers. The Nafion membrane is soaked in an aqueous sulfuric acid solution (pH = 0) for at least 48 hours, followed by three

rinses with ultra-pure water before use. Each chamber is equipped with an inlet and an outlet for the electrolyte, with a flow rate of 20 mL min^{-1}. CO_2 gas is supplied to the gas chambers at a flow rate of 50 sccm. Ir@Ti mesh serves as the counter electrode. The reference electrode (Ag/AgCl saturated KCl electrode) is inserted in the catholyte chamber. The potential of the working electrode versus SHE is calculated according to:

$$E \text{ (versus SHE)} = E \text{ (versus Ag/AgCl)} + 0.197 \text{ V} + iR \qquad (1)$$

R values are obtained between working and reference electrode from electrochemical impedance spectroscopy (EIS), which are measured at open circuit potential with the frequency range from 100 kHz to 0.1 Hz.

Electrochemical measurements in MEAs. In acidic MEA experiments, the cathode and anode chambers are separated by a Nafion 115 cation-exchange membrane. The cathode catalyst, either CoPc@CNT or CoPc@CNT-NH_2, enables the electrochemical reduction of CO_2 to CO, while the anodic catalyst, Ir@Ti mesh, facilitates water oxidation. For the cathodic compartment, CO_2 gas is introduced into the cathode chamber at a flow rate of 50 sccm for conversion. Prior to the reaction, the CO_2 gas is humidified by passing through a water-filled humidifying bottle, maintaining its moisture content. This humidification step prevents the CO_2 gas from dehydrating the cathode, thereby minimizing unwanted salt precipitation. Meanwhile, an H-type cell is employed to support the NH_3/NH_4^+ recirculation. The two compartments of the H-type cell serve distinct roles. One functions as the "NH_3 adsorption container" and the other as the "NH_4^+ supply container". Both compartments contain an identical solution consisting of H_2SO_4 and $(NH_4)_2SO_4$ in varying molar ratios. These compartments are separated by a gas-liquid separation membrane (Nafion 117), which prevents gas mixing between the cathode and anode while allowing cation transport. During electrochemical operation, NH_4^+ and H^+ ions from the "NH_4^+ supply container" are delivered to the anodic region of the MEA using a peristaltic pump. Under the electric field, these ions migrate to the cathode region, enhancing CO_2 reduction. At the cathode, NH_4^+ releases protons to form NH_3, which subsequently reacts with CO_2 and H_2O to produce NH_4HCO_3 precipitates. Upon heating, this precipitate decomposes, releasing NH_3 gas. The NH_3 gas is then transferred to the "NH_3 adsorption container", where it is protonated by H^+ ions and reabsorbed, regenerating NH_4^+. Through diffusion, the NH_4^+ returns to the "NH_4^+ supply container", establishing a continuous NH_3/NH_4^+ recirculation cycle.

In long-term stability experiments, we adopt an operational protocol comprising 10-hour reaction cycles

interspersed with 1-hour pauses. During these intervals, we refill the humidification bottle with water to compensate for losses, ensuring that the CO_2 remains consistently humidified throughout the testing period.

Product detection. The electrodes are evaluated in potentiostatic (or galvanostatic) tests to determine the Faradaic efficiencies at various potentials (or current densities). Electrochemical data is recorded every 12 minutes, and the gaseous products are subsequently analyzed using a gas chromatograph (Shimadzu, GC 2014C) equipped with flame ionization and thermal conductivity detectors.

COMSOL simulations. The one-dimensional generalized modified Poisson-Nernst-Planck (GMPNP) model is developed using COMSOL Multiphysics version 6.2, and the General Form PDE module within COMSOL is used to build the size dependent Nernst Planck equation and the Poisson equation[1]. In the simulation, it is essential to account for the combined effects of electric field, diffusion, convection, and chemical reactions. Despite the complexity, the system must obey the law of mass conservation:

$$\frac{\partial C_i}{\partial t} = -\nabla J_i + R_i \qquad (2)$$

Here, C_i represents the concentration of ionic species *i* in solution, including H^+, SO_4^{2-}, OH^-, NH_4^+ and K^+. J_i denotes the flux of species *i*, and R_i is its production rate. The ionic flux J_i incorporates contributions from diffusion, electromigration, and convection, and is expressed as:

$$\vec{J_i} = -D_i C_i \nabla\left(ln(\gamma_i C_i)\right) - \frac{D_i C_i z_i F}{RT}\nabla\varphi + \vec{v_x} C_i \qquad (3)$$

In this equation, the first term $-D_i C_i \nabla\left(ln(\gamma_i C_i)\right)$ represents diffusion influenced by ionic activity, the second term $-\frac{D_i C_i z_i F}{RT}\nabla\varphi$ accounts for electromigration under the electric field, and the third term $\vec{v_x} C_i$ describes convective transport induced by flow. The activity coefficient γ_i is modeled using a Langmuir-type expression to reflect ion crowding effects:

$$\gamma_i = \frac{1}{1 - N_A \sum_{i=1}^{N} a_i^3 C_i} \qquad (4)$$

Substituting Equation 4 into Equation 3 yields the expanded flux expression:

$$\vec{J_i} = -D_i \nabla C_i - D_i C_i \frac{N_A \sum a_i^3 \nabla C_i}{1 - N_A \sum a_i^3 C_i} - \frac{D_i C_i z_i F}{RT}\nabla\varphi + \vec{v_x} C_i \qquad (5)$$

Where D_i is the diffusion coefficient of species i, z_i is the charge number, F is Faraday's constant, R is

the universal gas constant, T is the temperature, φ is the electric potential (vs. PZC), N_A is Avogadro's number, and a_i is the effective hydrated diameter of species i. The axial velocity $\overrightarrow{v_x}$, resulting from the rotation of the disk electrode, is estimated by:

$$\overrightarrow{v_x} = -0.51x^2\sqrt{\omega^3/_v} \qquad (6)$$

Where ω is the angular velocity of the RDE and v is the kinematic viscosity of water.

Additionally, the water self-ionization equilibrium in the bulk phase is considered:

$$\mathrm{H_2O} \rightleftharpoons \mathrm{H^+} + \mathrm{OH^-} \qquad (7)$$

With forward and reverse rate constants denoted by k_{w1} (dissociation) and k_{w2} (recombination), respectively.

To account for the shielding effect caused by excess ionic charge, the electric potential distribution is described by the Poisson equation:

$$\nabla \cdot (-\varepsilon_0\varepsilon_r\nabla\varphi) = F\sum(z_iC_i) \qquad (8)$$

At the electrode interface (left boundary, x=0), both CO_2R and HER consume the species of H^+. Under H^+-rich conditions, although NH_4^+ and H_2O can act as proton donors, the produced NH_3 and OH^- readily react with excess H^+ to regenerate NH_4^+ and H_2O. Consequently, the net proton consumption is effectively attributed to H^+. Accordingly, the H^+ flux at x=0 is

$$\vec{J}_{\mathrm{H^+},x=0} = \frac{j_{\mathrm{H^+}}}{F} \qquad (9)$$

Where $j_{\mathrm{H^+}}$ is the current density of H^+ consumption，taking the values 50, 100, 150 and 200 mA cm^{-2}.

In COMSOL simulations, the geometry consists of a 1-dimensional domain extending from the outer Helmholtz plane (OHP) to a distance of 100 μm into the solution. The cation effect on localized H^+ concentration is investigated in two electrolyte systems: 0.1 M H_2SO_4 & 0.5 M $(NH_4)_2SO_4$, and 0.1 M H_2SO_4 & 0.5 M K_2SO_4. To simplify the simulations, the acid-base neutralization process between CO_2 and the reaction interface is excluded. The model setup follow previous studies[1], with parameters listed in Table S1. When simulating the effect of NH_4^+ cations on the localized H^+ concentration, the focus is placed on cases

where pH < 8. In this pH range, NH_3 rapidly acquires H^+ from the solution, forming NH_4^+. At pH > 8, the localized H^+ concentration becomes very low, rendering its impact on HER kinetics negligible.

DFT calculations. DFT calculations are performed using the Vienna Ab-Initio Simulation Package (VASP) with RPBE functional.[2] The core electrons are described with the projector augmented wave (PAW) method.[3,4] The convergence criteria for electronic and force minimization are set to 10^{-6} eV and 0.02 eV $Å^{-1}$ for the structure optimization. The cutoff energy for the kinetic energy of the plane-waves is 450 eV. The entropy corrections determined from frequency calculations are performed by using the harmonic oscillator approximation. Γ k-point is sampled for CoPc@CNT. The self-consistent implicit solvation model VASPsol[5] is used to represent the polarizable electrolyte region. The dielectric constant 78.3, and the Debye screening length 3 Å, are used, as it corresponds to a bulk ion concentration of 1 M. For NH_4^+, we used NH_4^+ with 6 water molecules surroundings, namely, the water cluster model to represent the local solvation environment, and the implicit solvent model presents the rest.

This electric field distribution is obtained via the following equation[6]:

$$\varepsilon = \frac{d(V_{env} - V_{slab} - V_{solvent} - V_{ion})}{dz} \qquad (10)$$

where ε represents electric field, $V_{env}, V_{slab}, V_{solvent}$ and V_{ion} are the electrostatic potential of the environment, the slab, the solvent, and the ion respective.

Molecule calculations are performed using the Gaussian 16 program. The geometry optimizations are performed using B3LYP functional[7] with def2-TZVP basis sets[8] and D3 correction[9] (Becke–Johnson damping) to better account for the dispersion interactions. Solvent effects are modelled using the implicit SMD model.[10] The Gibbs free energy of proton is derived using thermodynamics and the Sackur-Tetrode equation, giving $H^0(H^+)$ = 5/2 RT = 1.48 kcal mol^{-1} and S^0 = 26.5 cal/(mol K) at 298.15 K and 1 atm.

Energy Consumption and Operating Cost Estimation

(1) Energy consumption estimation: the energy consumption to produce 1 mole of CO is divided into 5 parts: equilibrium, voltage loss, heat, side reaction and gas separation.

Equilibrium corresponds to the change of Gibbs free energy of the overall reaction. It was calculated according to:

$$\Delta G_m = 2FU_{\text{equilibrium}} \qquad (11)$$

$$U_{\text{equilibrium}} = \varphi_{H_2O/O_2} - \varphi_{CO/CO_2} \qquad (12)$$

where F is the Faradaic constant (96485 C mol^{-1}), $U_{\text{equilibrium}}$ is the thermodynamic equilibrium voltage, $\varphi_{\text{H2O/O2}}$ is the equilibrium electrode potential for OER (1.23 V vs RHE) and $\varphi_{\text{CO/CO}_2}$ is the equilibrium electrode potential for CO_2 reduction (-0.11 V vs RHE for CO formation) at standard conditions. Therefore, the equilibrium term is 258.6 kJ mol^{-1} for CO formation.

Voltage loss is due to the overpotential of CO_2 reduction and OER and ohmic loss. The energy consumption corresponding to the difference between the applied voltage (U_{appiled}) and $U_{\text{equilibrium}}$ is collectively referred to as voltage loss:

$$\Delta E_{\text{voltage}} = 2F(U_{\text{appiled}} - U_{\text{equilibrium}}) \quad (13)$$

Heat corresponds to the energy consumption required for the heating up and heat dissipation during the operation of our MEA electrolyzer. It is calculated according to:

$$\Delta E_{\text{heat}} = \Delta E_{\text{heat1}} + \Delta E_{\text{heat2}} \quad (14)$$

ΔE_{heat1} is the energy consumption for heating up from room temperature (25 ℃) to the operating temperature (60 ℃):

$$\Delta E_{\text{heat1}} = V\rho c(60\,°\text{C} - 25\,°\text{C}) \quad (15)$$

where V is the volume of Ti MEA we used ($7.5 \times 7.5 \times 4$ cm^3), ρ is the density of Ti metal (4.505 g cm^{-3}), and c is the specific heat capacity of Ti (0.52 J/(g ·℃)).

ΔE_{heat2} is the energy consumption of heat dissipation loss at 60 ℃:

$$\Delta E_{\text{heat1}} = \frac{Akt}{L}(60\,°\text{C} - 25\,°\text{C}) \quad (16)$$

$$t = \frac{2F}{\text{FE\%} \times 100\ \text{mA}} \quad (17)$$

where A is the surface area of the MEA we used ($(7.5 \times 7.5 + 7.5 \times 4 + 7.5 \times 4) \times 2$ cm^2), k is the thermal conductivity of the insulation layer for heat preservation (0.0015 W/(m ·K),[11] L is the thickness of the insulation layer (0.04 m), t is the operating time for producing 1 mole of CO, FE% is the Faradaic efficiency of CO.

Side reaction corresponds to the energy consumption used for the production of side product (H_2) as 1 mole of CO forms, and is calculated according to:

$$\Delta E_{\text{side}} = (\Delta G_m + \Delta E_{\text{voltage}})\frac{100\% - \text{FE\%}}{\text{FE\%}} \quad (18)$$

Gas separation corresponds to the energy consumption needed to separate CO_2 from the gas mixture from the cathode and the anode. Commercially mature monoethanolamine-based scrubbing process is used for the

separation and the energy consumption is 179 kJ/mol$_{CO2}$.[12] Unreacted CO_2 is calculated based on single-pass conversion efficiency (SPCE):

$$n_{\mathrm{unreated}} = 1\ \mathrm{mol} \times \frac{100\% - \mathrm{SPCE}\%}{\mathrm{SPCE}\%} \qquad (19)$$

$$\mathrm{SPCE}\% = \frac{100\ \mathrm{mA} \times \mathrm{FE}\% \times RT}{2Fvp} \qquad (20)$$

where R is the gas constant (8.314 J/(mol K)), T is the temperature at the gas outlet, v is the flow rate at the gas outlet, and p is the pressure at the gas outlet.

Thus, to produce 1 mole of CO, the energy consumption for gas separation is:

$$\Delta E_{\mathrm{separation}} = n_{\mathrm{unreated}} \times 179\ \mathrm{kJ/mol_{CO_2}} \qquad (21)$$

(2) Operating cost estimation: The thermal energy price is assumed to be \$2.1 per million British Thermal Units[13], and the electricity price is assumed to be \$0.04 per KWh[14]. Heating and gas separation utilize thermal energy, while the rest consume electrical energy.

Supplementary Figures

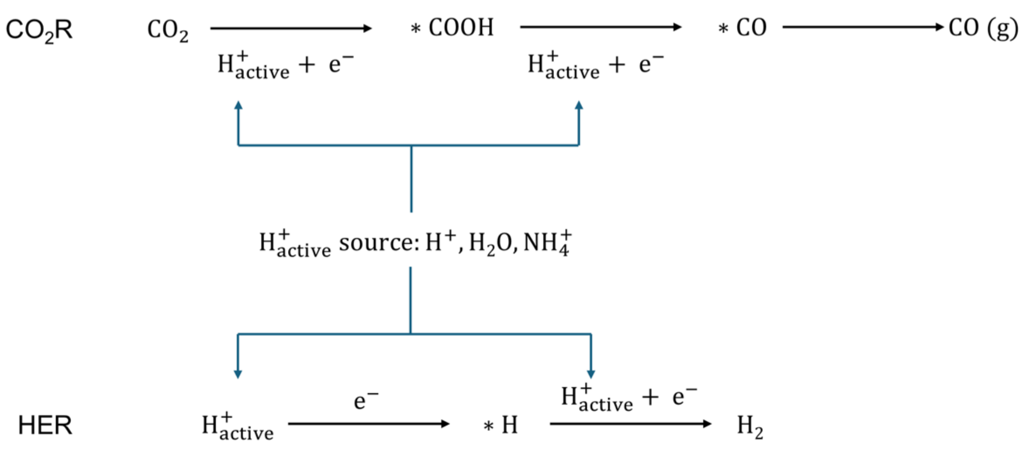

Figure S1. The source of H^{+}_{active} and its role in the CO_2R and HER pathways.

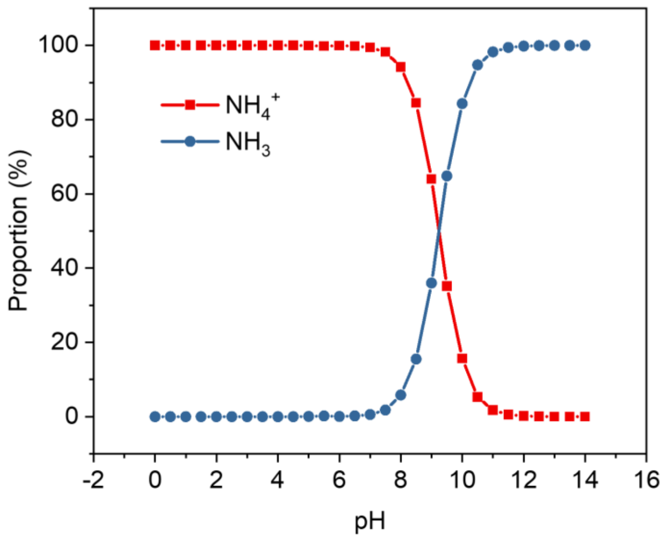

Figure S2. Proportional distribution of NH_4^+ and NH_3 at different pH values.

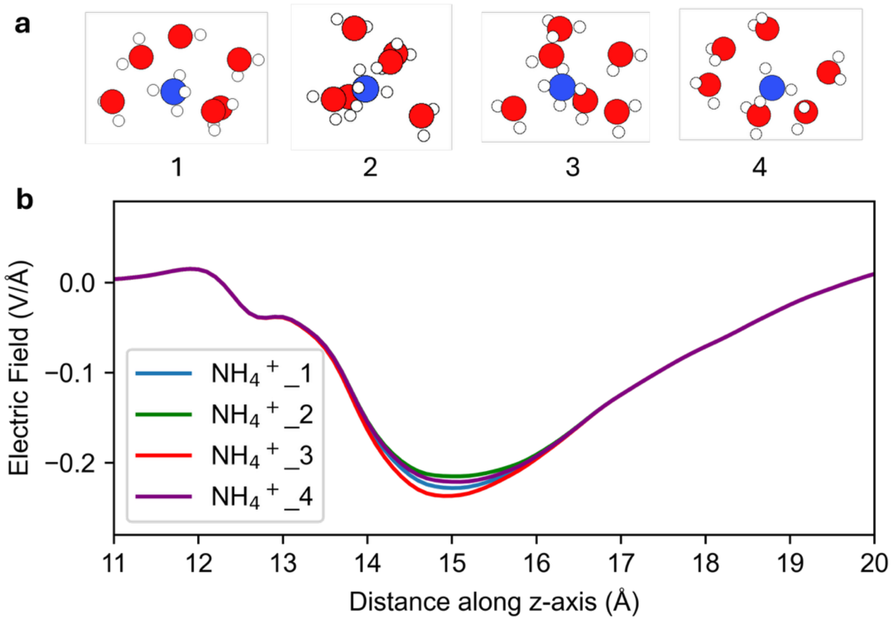

Figure S3. (a) The water cluster structures chosen from Ab initio molecular dynamics (AIMD, slab is shown in the picture). (b) Averaged electric field distribution along the z-axis near the catalyst surface under different solvent structures.

Even as individual water molecules in the $NH_4^+ (H_2O)_6$ cluster reorient on the picosecond timescale, the computed vertical electric field at the CoPc@CNT interface remains remarkably constant, with fluctuations of less than 0.03 V $Å^{-1}$ over four randomly sampled frames from a 5 ps AIMD trajectory. These small variations underscore that the dominant contribution to the interfacial field arises from net charge transfer between NH_4^+ and the surface rather than from transient changes in hydrogen-bonding geometry. Because the dipole vectors of the solvent shell arrange approximately symmetrically around the cation, secondary field components largely cancel out, validating our use of a six-water microsolvation cluster to represent the first solvation shell. Consequently, this model captures the key physics governing the field-dipole interaction for CO_2 adsorption, without the prohibitive cost of an explicit water layer. Although a fully explicit solvent model with precise electrode-potential control would yield more realistic solvation structures, it is computationally demanding and thus represents a valuable direction for future investigations.

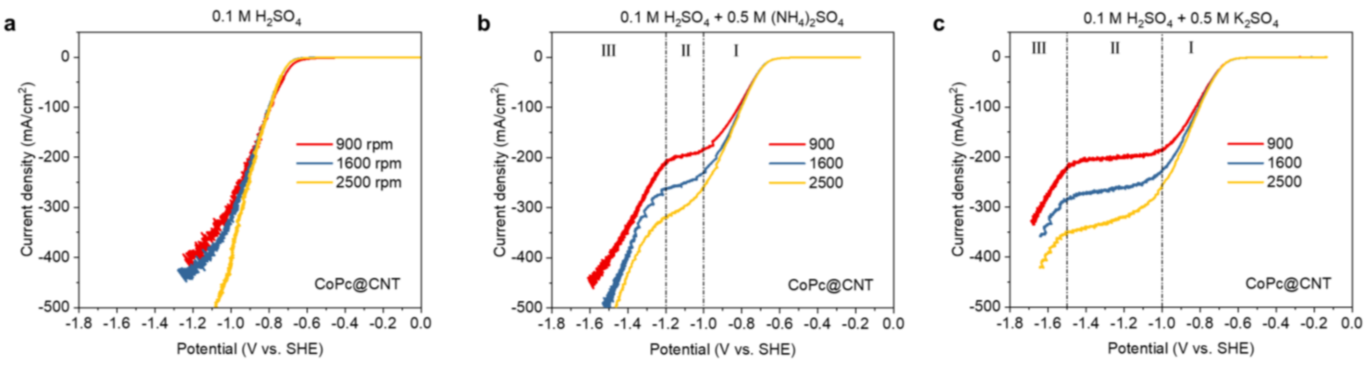

Figure S4. Polarization curves of CoPc@CNT catalysts in (a) 0.1 M H_2SO_4, (b) 0.1 M H_2SO_4 and 0.5 M $(NH_4)_2SO_4$, and (c) 0.1 M H_2SO_4 and 0.5 M K_2SO_4 at various rotation speeds (900, 1600, and 2500 rpm) using RDE.

In the systems with added cations (0.1 M H_2SO_4 + 0.5 M K_2SO_4 and 0.1 M H_2SO_4 + 0.5 M $(NH_4)_2SO_4$), the presence of K^+ and NH_4^+ inhibits the electromigration of H^+ toward the electrode surface, resulting in H^+ transport limitations. In contrast, for the 0.1 M H_2SO_4 system without additional cations, the observed H^+ transport limitation arises from a different mechanism. Under these conditions, a high rate of H_2 evolution leads to significant gas bubble accumulation on the electrode surface. At rotation rates of 900 and 1600 rpm, these bubbles are not effectively removed, which blocks access of H^+ to the surface and causes mass transport limitations. When the rotation speed is increased to 2500 rpm, bubble removal becomes sufficient and the limitation is eliminated. Thus, while both cases exhibit H^+ transport limitations, the underlying mechanisms differ: in the cation-containing systems, it is due to suppressed H^+ migration, whereas in the cation-free system, it is caused by bubble-induced physical blockage.

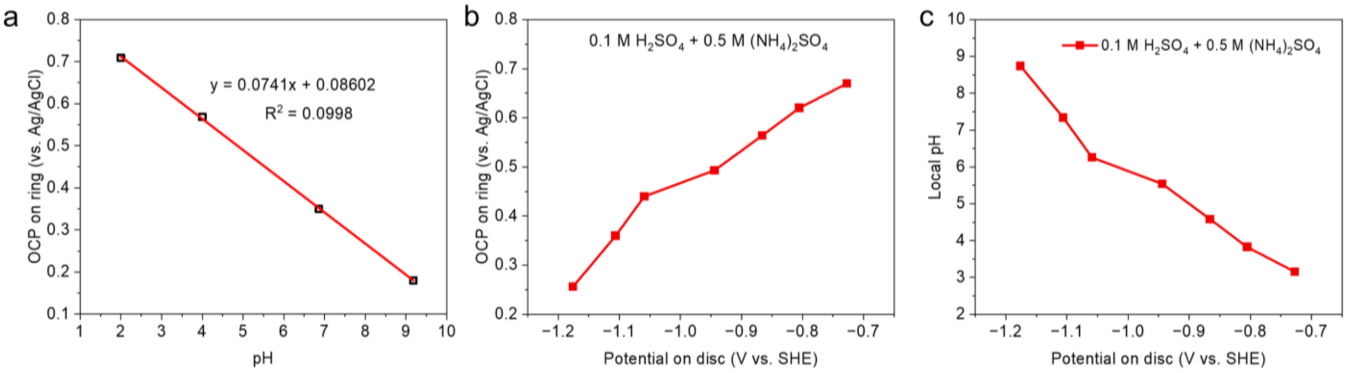

Figure S5. Local pH analysis in 0.1 M H_2SO_4 + 0.5 M $(NH_4)_2SO_4$ using a rotating ring-disk electrode (RRDE). (a) Calibration curve between OCP on the IrO_2 ring electrode and pH. (b) OCP on the IrO_2 ring at varying disk potentials. (c) Derived local pH values near the disc surface based on the calibration.

We employ a rotating ring-disk electrode (RRDE) to probe the local pH. The open-circuit potential (OCP) of the IrO_2-coated ring electrode correlates with pH (Figure S5a), enabling in situ detection of the local pH in the electrolyte near the disk. This technique is widely reported in previous studies.[15–17] Based on this method, we measure the local pH in the NH_4^+-containing system (Figure S5b-c). The results show that the local pH gradually increases with more negative applied potentials, reaching a value of 8.7 at -1.2 V vs SHE, which is significantly higher than the bulk solution pH of 1.21. These findings experimentally demonstrate that NH_4^+ effectively increases the local pH during electrolysis.

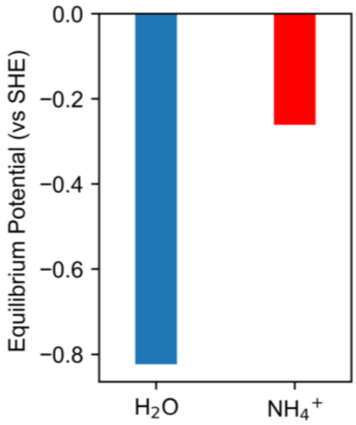

Figure S6. The calculated equilibrium potential for the electroreduction to CO using different proton sources on CoPc@CNT.

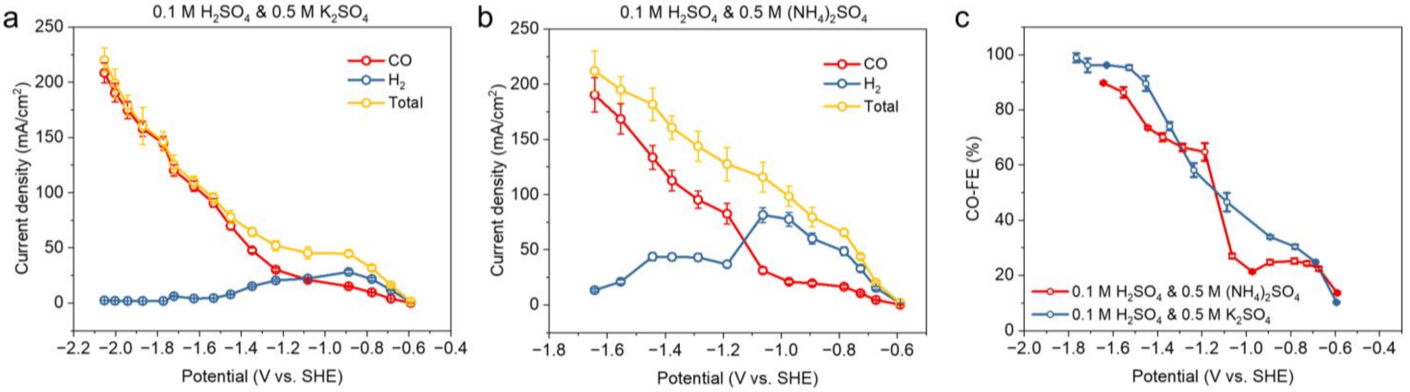

Figure S7. CO_2R performance in 0.1 M H_2SO_4 with either 0.5 M K_2SO_4 or 0.5 M $(NH_4)_2SO_4$. (a, b) Current densities of CO, H_2, and total products in 0 H_2SO_4 with 0.5 M K_2SO_4 or 0.5 M $(NH_4)_2SO_4$. (c) CO-FE under the two conditions. Error bars indicate standard deviations.

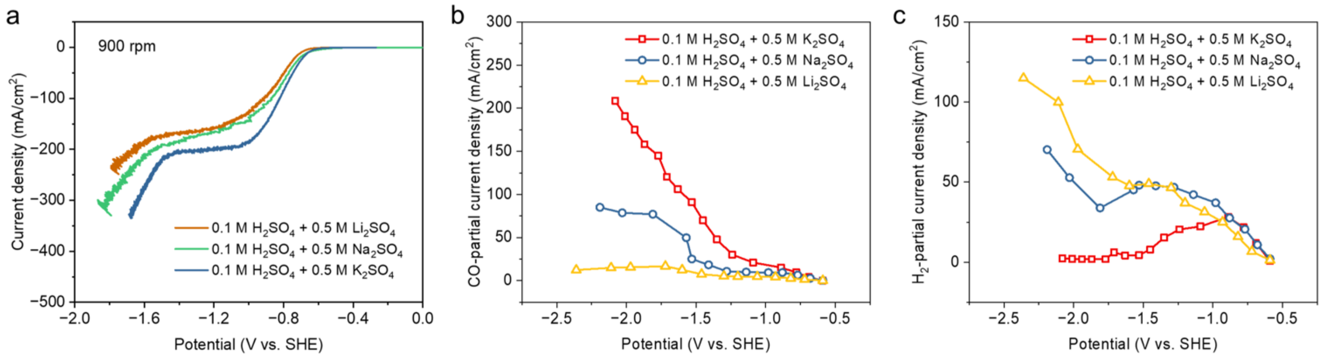

Figure S8. (a) HER polarization curves of CoPc@CNT catalyst in 0.1 M H_2SO_4 with 0.5 M Li_2SO_4, Na_2SO_4, or K_2SO_4 using RDE with 900 rpm. (b) CO partial current densities and (c) H_2 partial current densities as a function of applied potential for CO_2R in 0.1 M H_2SO_4 with 0.5 M Li_2SO_4, Na_2SO_4, or K_2SO_4 using flow cells.

RDE-based HER tests show that the polarization curves of Li^+ and Na^+ are similar to that of K^+, exhibiting three distinct regions. The presence of a plateau suggests that, like K^+, Li^+ and Na^+ can suppress the migration of H^+ to the electrode surface. At the point of H^+ depletion, the onset potentials for water reduction in Li^+, Na^+, and K^+ systems are similar and more negative than that in the NH_4^+ system, indicating that NH_4^+ exhibits a stronger proton-donating capability. Furthermore, CO_2R performance in the presence of Li^+ and Na^+ is inferior to that with K^+, favoring HER over CO_2R, which is consistent with previous reports.[18–20] This can be attributed to the much lower solubility of Li^+- and Na^+-based bicarbonates compared to K^+-based bicarbonates. Upon H^+ depletion, the local pH increases, promoting severe bicarbonate precipitation under high-pH conditions. This precipitation hinders CO_2 transport to the catalyst surface. Therefore, the more negative CO_2 reduction potentials observed in Li^+ and Na^+ systems compared to the NH_4^+ system arise not only from the use of water as a weaker proton donor but also from the additional barrier caused by salt precipitation.

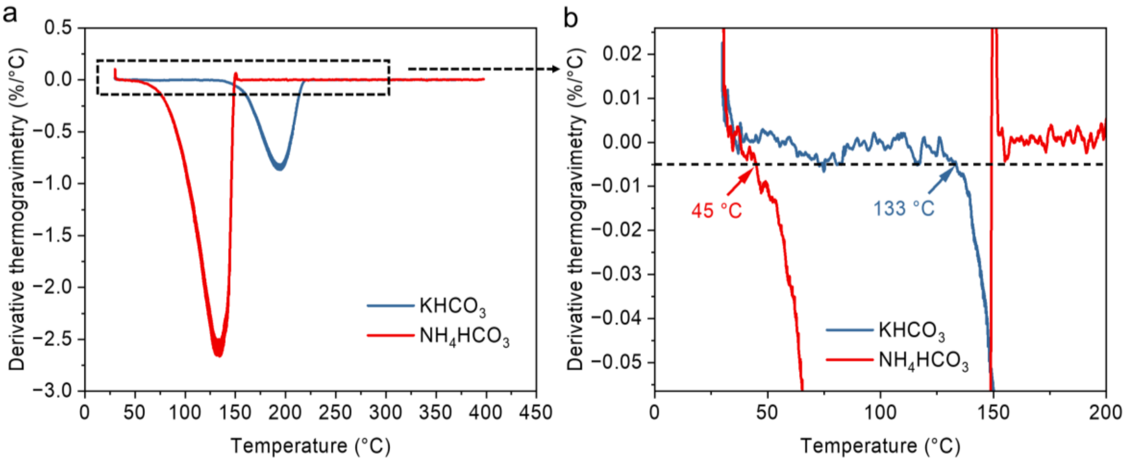

Figure S9. Decomposition behavior of $KHCO_3$ and NH_4HCO_3 analyzed by derivative thermogravimetry (DTG). (a) Full-range DTG curves. (b) Enlarged view highlighting the onset of decomposition, which occurs at 45 ℃ for NH_4HCO_3 and 133 ℃ for $KHCO_3$.

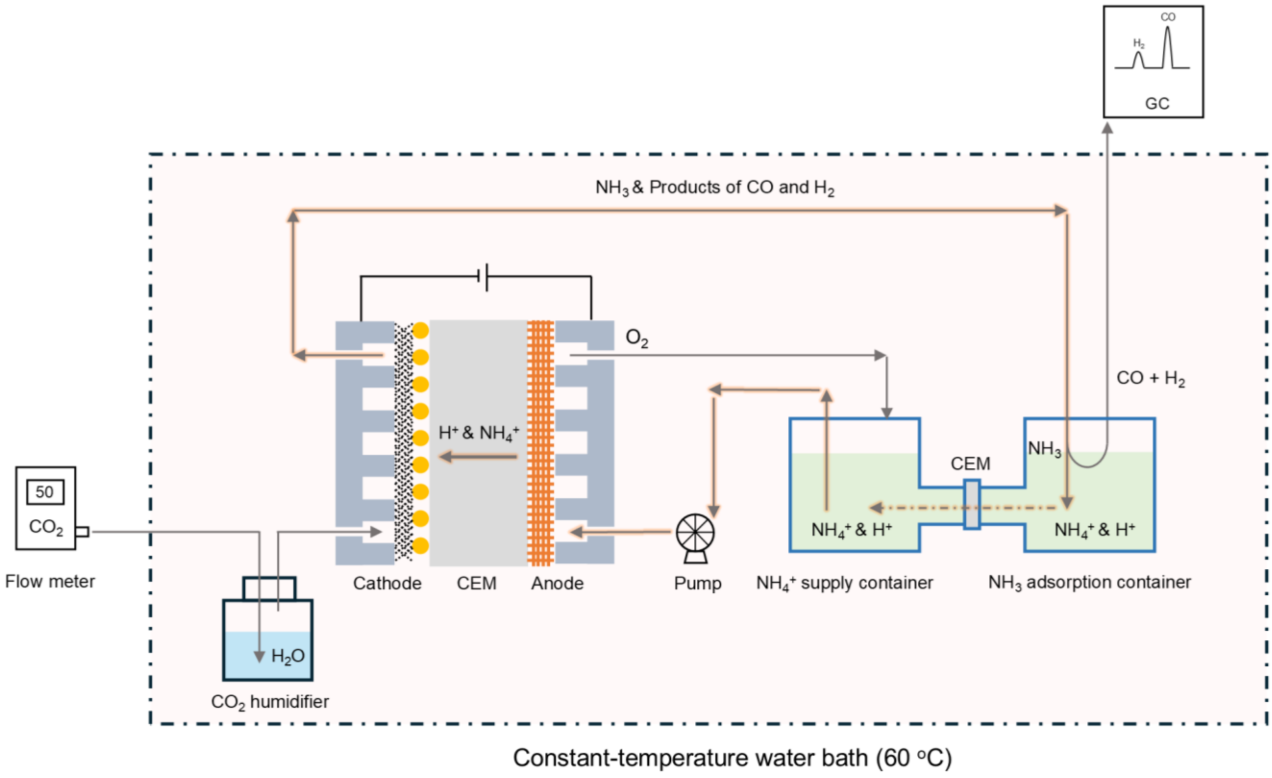

Figure S10. NH_3/NH_4^+ recirculation system constructed using an H-type cell and a constant-temperature water bath.

In our laboratory setup, we construct the NH_3/NH_4^+ recirculation system using an H-type electrolytic cell placed in a constant-temperature water bath. One compartment of the H-cell functions as the NH_4^+ supply container, providing NH_4^+ and H^+ for the MEA, while the other compartment serves as the NH_3 adsorption container, absorbing the NH_3 generated at the cathode and converting it back into NH_4^+. These two compartments are separated by a cation exchange membrane (Nafion 117), which not only allows for NH_4^+ and H^+ transport but also prevents gas crossover between the compartments. To provide the temperature necessary for NH_4HCO_3 decomposition, the entire system is placed in a thermostatic water bath.

Regarding scalability, the NH_3/NH_4^+ recirculation system can draw on the well-established infrastructure of industrial water electrolysis systems,[21–23] which typically operate at temperatures between 60-80 ℃. In scaled-up systems, the ohmic and overpotential losses from high current densities are converted into heat, which in turn helps maintain the required operating temperature. During the startup phase, external heating can be provided through heat exchangers, which can utilize low-grade industrial waste heat at minimal cost.

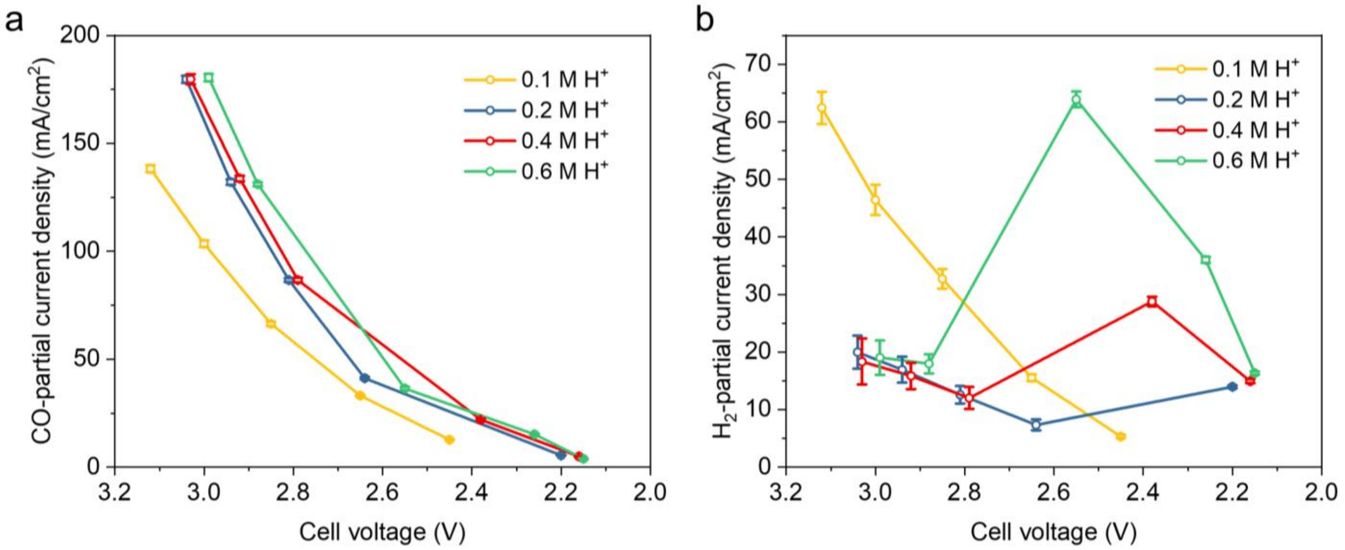

Figure S11. (a) CO partial current density, (b) H_2 partial current density in the anolytes containing 0.2 M NH_4^+ with varying concentrations of H^+.

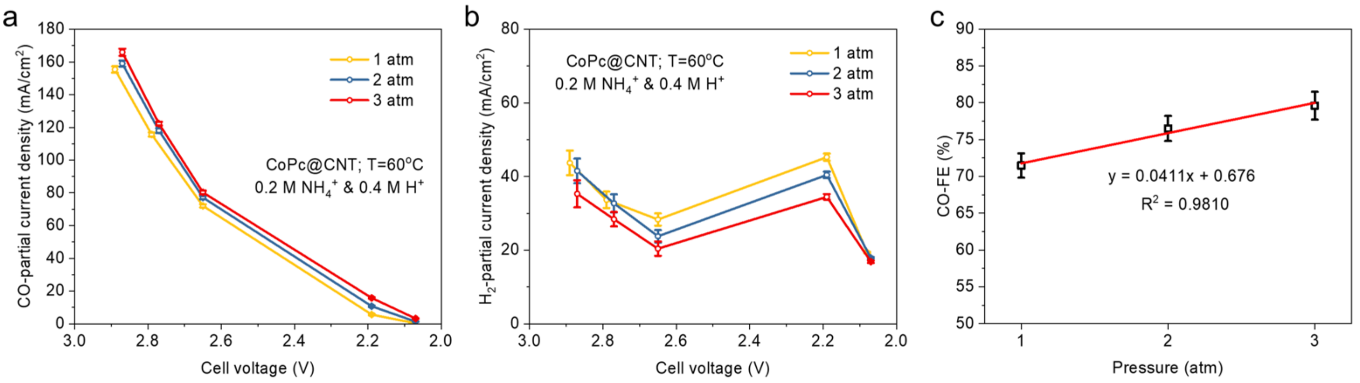

Figure S12. Pressure effect on CO_2R with CoPc@CNT at 60 °C in 0.2 M NH_4^+ and 0.4 M H^+. (a) CO partial current density, (b) H_2 partial current density, and (c) CO-FE under 1, 2, and 3 atm.

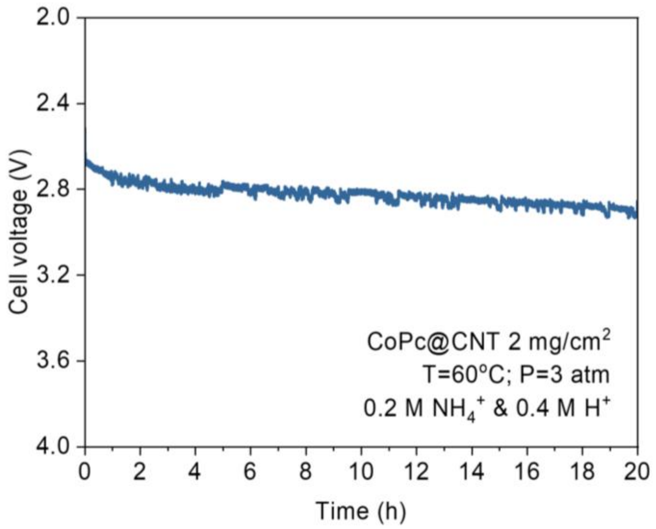

Figure S13. Cell voltage profile of CoPc@CNT during 20-hour MEA operation at 60 °C and 3 atm using the anolyte of 0.2 M NH_4^+ and 0.4 M H^+. Catalyst loading: 2 mg cm^{-2}.

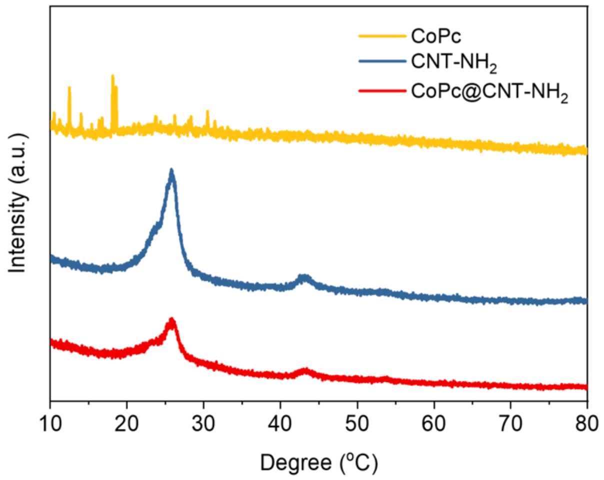

Figure S14. X-ray diffraction patterns of CoPc, $CNT\text{-}NH_2$, and $CoPc@CNT\text{-}NH_2$.

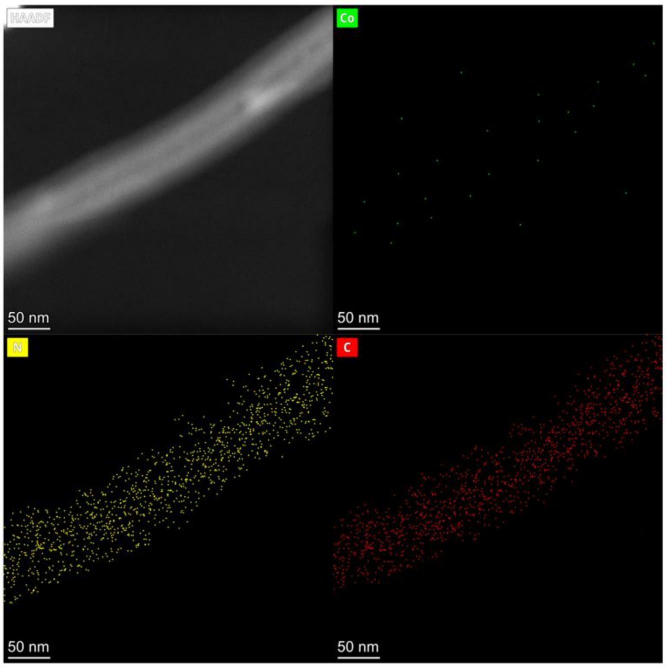

Figure S15. STEM-EDS elemental mapping of CoPc@CNT-NH_2. The HAADF image (top left) and corresponding elemental maps of Co (green), N (yellow), and C (red) confirm the uniform distribution of nitrogen and carbon along the nanotube.

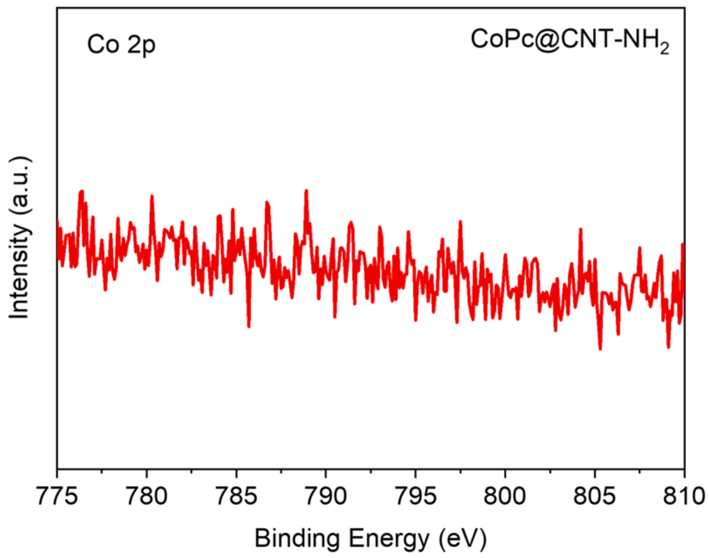

Figure S16. XPS spectrum of Co 2p region for CoPc@CNT-NH_2.

Since the synthesis method is similar to that of CoPc@CNT, the structure of CoPc@CNT-NH_2 is theoretically similar, with CoPc molecules expected to be molecularly dispersed on CNT-NH_2. The XRD pattern of CoPc@CNT-NH_2 shows only the characteristic signals of CNT-NH_2 (Fig. S14), with no detectable peaks corresponding to CoPc aggregates, indicating good dispersion. This is further supported by TEM images (Fig. S15), which confirm that Co species are well dispersed on CNT-NH_2 without noticeable aggregation. Moreover, due to the very low Co loading (0.25 ωt%), the Co 2p signal is not prominent in the XPS spectrum (Fig. S16). These results collectively demonstrate that CoPc is uniformly dispersed on CNT-NH_2 without aggregation.

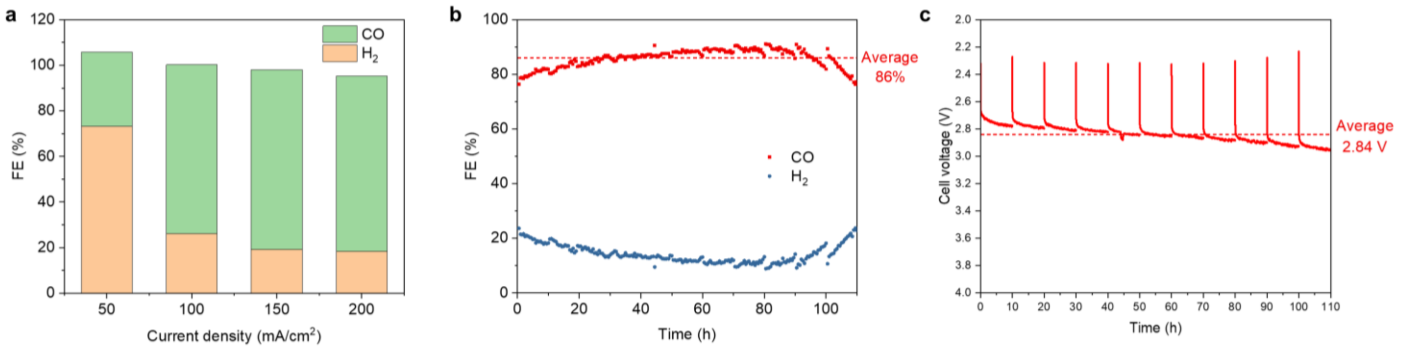

Figure S17. (a) Faradaic efficiencies of CO and H_2 production at different current densities for CoPc@CNT-NH_2 catalyst. (b) Long-term stability test showing Faradaic efficiencies of CO and H_2 production over 110 hours at 100 mA cm^{-2}. The average CO selectivity maintains at 86% throughout the test period. (c) Cell voltage profile during the 110-h stability test, showing an average voltage of 2.84 V. Testing conditions: 0.2 M H_2SO_4 and 0.1 M $(NH_4)_2SO_4$ anolyte at 60 ℃ and 3 atm.

The fluctuation between 40 and 50 hours in cell voltage is attributed to a temporary disturbance in the laboratory environment. An unexpected change in indoor air pressure generated a strong airflow directed toward the electrochemical setup, which caused a drop in the reaction temperature. This temperature decrease led to a corresponding rise in cell voltage. Once the airflow interference was eliminated, the reaction temperature returned to normal, and the cell voltage stabilized accordingly. Therefore, the fluctuation observed between 40 and 50 hours results from this temporary environmental perturbation rather than any intrinsic instability of the system.

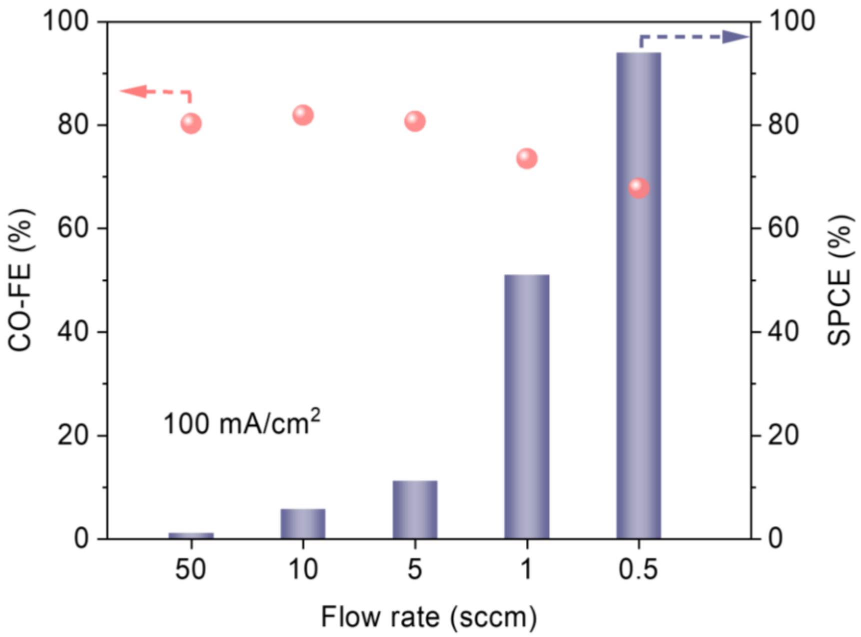

Figure S18. Effect of flow rate on CO-FE (left axis) and single-pass conversion efficiency (SPCE, right axis) at a current density of 100 mA cm^{-2} on CoPc@CNT-NH_2.

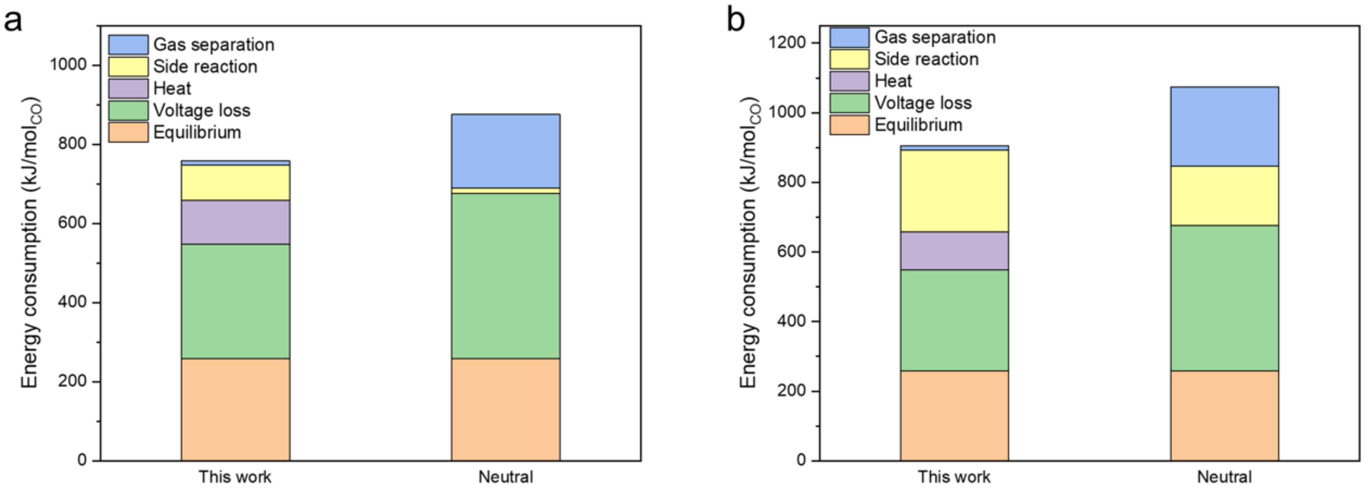

Figure S19. Estimation of energy consumption required to produce 1 mole of CO (a) under ideal conditions and (b) quasi-realistic conditions. The calculation under neutral conditions is based on performance parameters reported in Hao's work.[24]

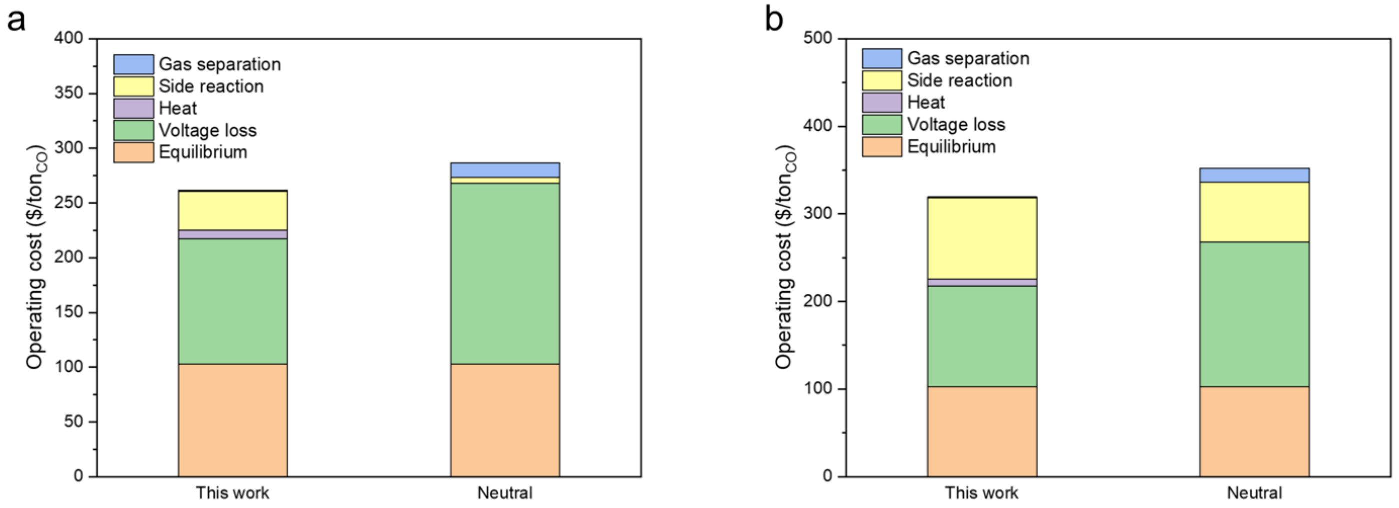

Figure S20. Estimation of operating cost required to produce 1 ton of CO (a) under ideal conditions and (b) quasi-realistic conditions. The calculation under neutral conditions is based on performance parameters reported in Hao's work.[24]

We have incorporated a comprehensive comparison with traditional neutral CO_2R systems. We chose not to compare with alkaline systems due to their significant electrolyte regeneration costs, as noted by Gu's work[25]. Instead, we compared our system against leading neutral systems (Table S3) reported by Hao[24], which is recognized as the current state-of-the-art in neutral CO_2R systems. Our analysis evaluated two scenarios: ideal conditions, where both CO-FE and SPCE are optimized, and quasi-realistic conditions, where higher SPCE results in an unavoidable reduction in CO-FE (Table S4). In both scenarios, our system demonstrates lower energy consumption (Figure S19) and operational costs (Figure S20) compared to Hao's system, driven by its superior cell voltage and higher SPCE. In Hao's work, a low-concentration anolyte (0.01 M $KHCO_3$) is used to suppress salt precipitation and improve stability; however, this approach increases ohmic losses, resulting in lower energy efficiency and higher cell voltage (Table S3). Furthermore, neutral systems generally suffer from CO_2 crossover through the membrane, which leads to a lower SPCE. Additionally, the energy required for NH_4HCO_3 heating is minimal relative to these efficiency gains. These findings, along with detailed energy and cost comparisons, are presented in Figure S19 and Figure S20.

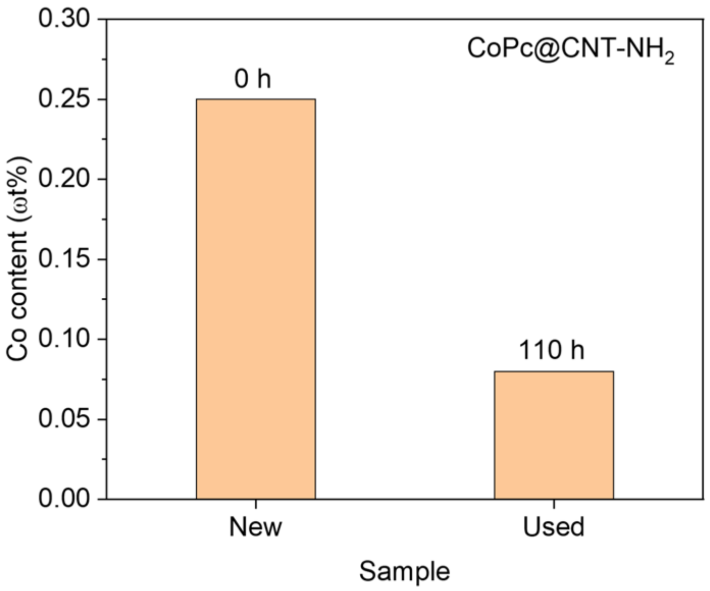

Figure S21. Comparison of cobalt content in CoPc@CNT-NH_2 before and after electrolysis.

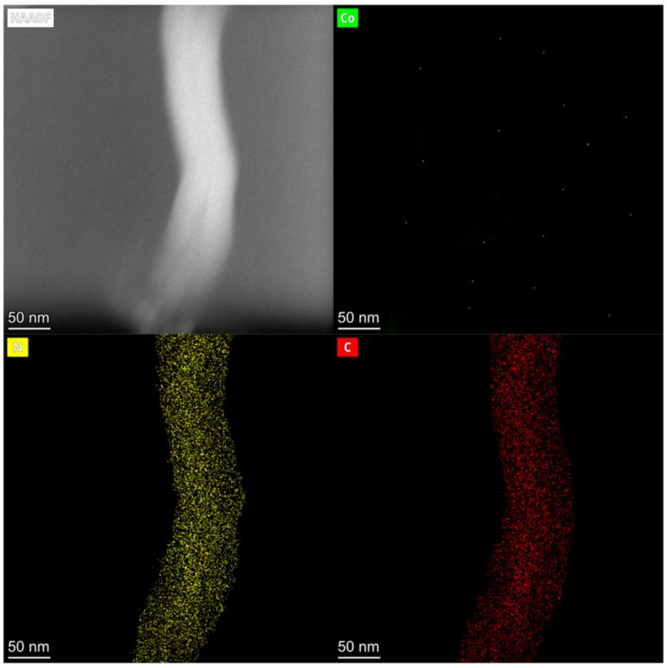

Figure S22. STEM-EDS elemental mapping of CoPc@CNT-NH_2 after 110 hours of electrolysis. The HAADF image (top left) and elemental maps of Co (green), N (yellow), and C (red).

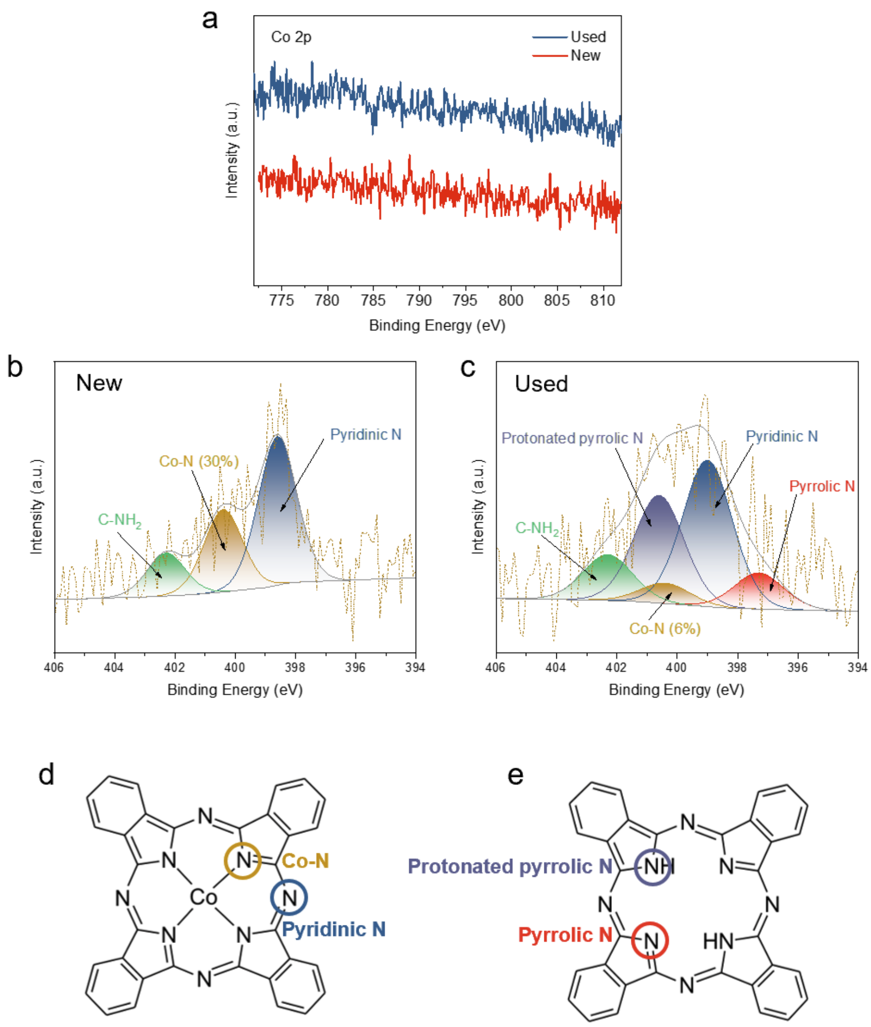

Figure S23. XPS analysis of CoPc@CNT-NH_2 before and after electrolysis. Comparison of (a) Co 2p and (b, c) N 1s spectra of the new and used samples. The new sample shows dominant Co-N and pyridinic N, while the used sample displays reduced Co-N with increased pyrrolic and protonated pyrrolic N. (d, e) N species in the CoPc and H_2Pc molecule: (d) pyridinic N and Co-N in CoPc molecule; (e) protonated and unprotonated pyrrolic N in H_2Pc molecule.

Owing to the low Co loading, the Co 2p signal in the XPS spectrum is weak and not well resolved. The leaching of Co is accompanied by proton coordination, which alters the local chemical environment of nitrogen species during electrolysis. As a result, the relative proportion of Co-N species decreases, while the fractions of protonated and unprotonated pyrrolic nitrogen increase.

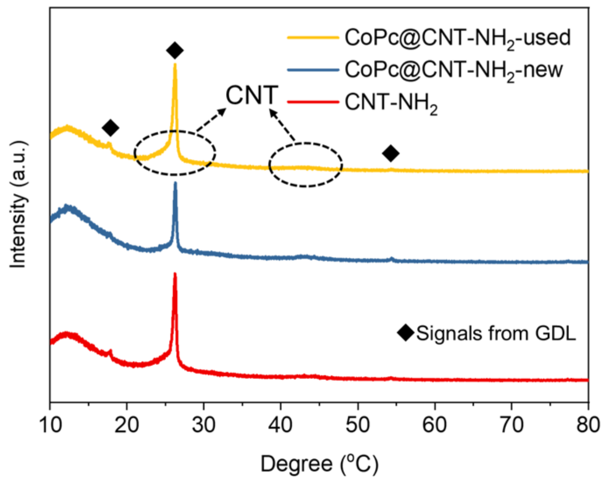

Figure S24. XRD patterns of $CNT\text{-}NH_2$, new $CoPc@CNT\text{-}NH_2$, and used $CoPc@CNT\text{-}NH_2$ after electrolysis.

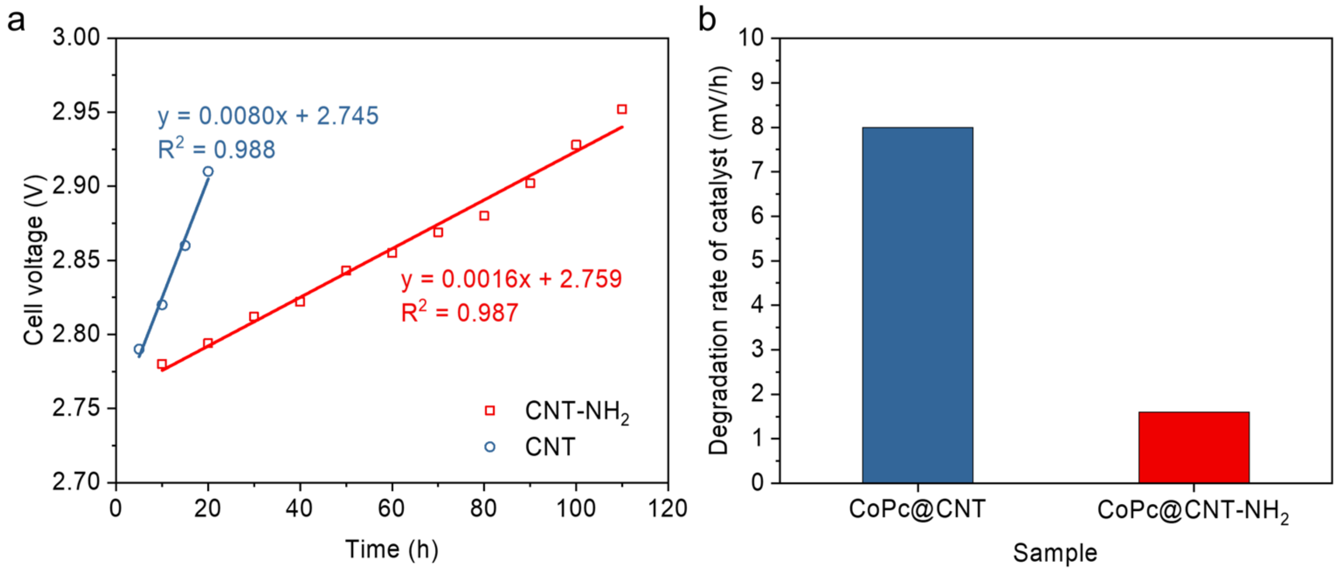

Figure S25. (a) Cell voltage profiles over time and (b) degradation rates of CoPc@CNT and CoPc@CNT-NH_2 under the electrolysis.

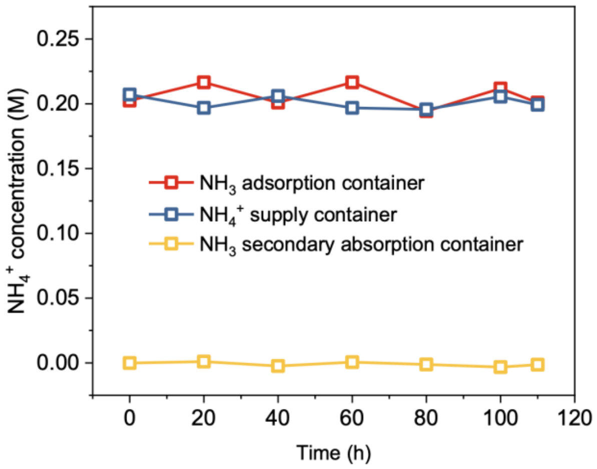

Figure S26. NH_4^+ concentration in different containers over 110 hours of operation. The NH_3 adsorption container and NH_4^+ supply container maintain stable NH_4^+ concentrations around 0.2 M, while the NH_3 secondary absorption container (0.1 M H_2SO_4) shows negligible accumulation.

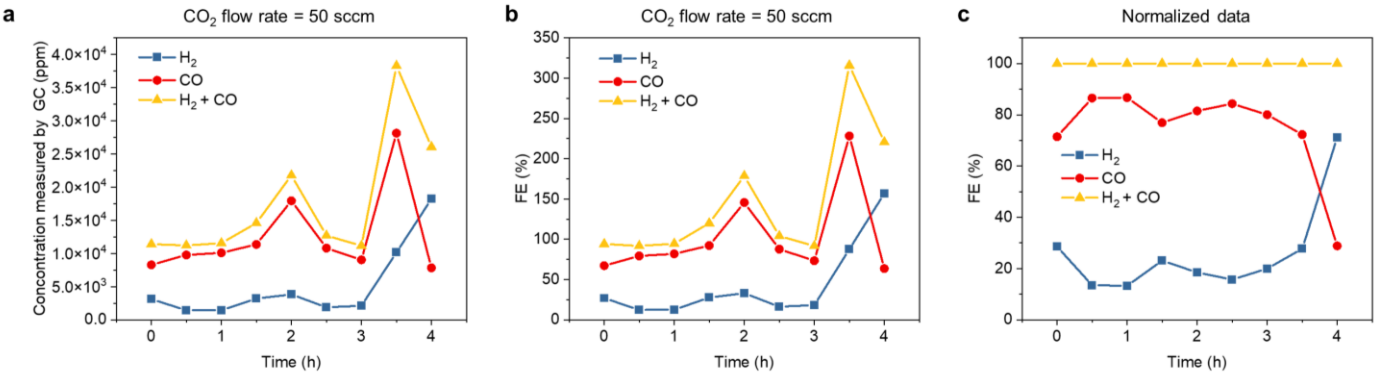

Figure S27. Test data of the electrochemical CO_2R process on CoPc@CNT-NH_2 in 0.2 M H_2SO_4 and 0.1 M $(NH_4)_2SO_4$ anolyte at 60 ℃ and 3 atm. (a) Concentrations of H_2, CO, and their sum (H_2 + CO) over time, as measured by gas chromatography. (b) Faradaic efficiency of H_2, CO, and their combined values (H_2 + CO) as a function of time. (c) Normalized Faradaic efficiency data showing the proportions of H_2, CO, and total products (H_2 + CO) over time.

We calculated the Faradaic efficiency of CO and H_2 based on their concentrations measured by gas chromatography. However, during the experiments, we frequently observed that the combined Faradaic efficiency of CO and H_2 exceeded 100%. This anomaly was primarily attributed to fluctuations in the measured concentrations of CO and H_2, caused by salt precipitation within the system (Figure S10). The salt precipitation disrupted the gas flow, resulting in deviations of the actual flow rate from the set value of 50 sccm (Figure S9), which subsequently led to overestimation in the calculated values. To address this issue, we adopted a normalization approach, where the combined Faradaic efficiency of CO and H_2 was constrained to 100% to provide more accurate and reliable results.

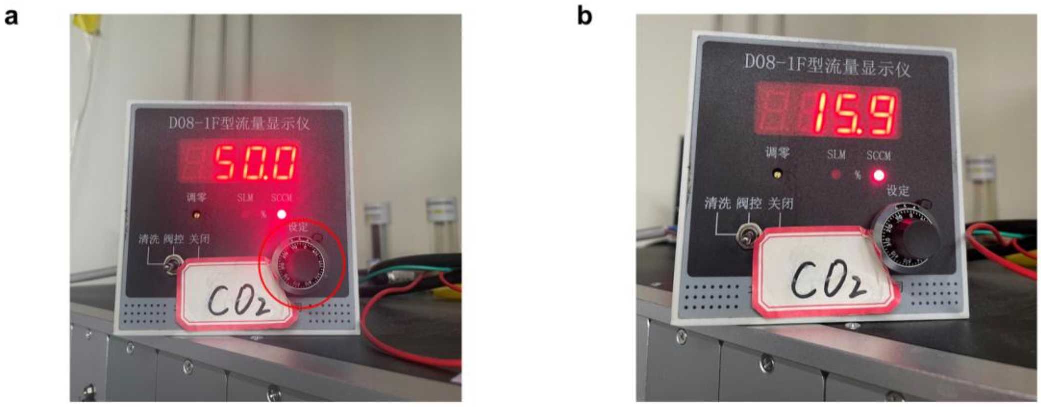

Figure S28. Flow meter readings for CO_2 gas supply during the electrochemical CO_2R experiments on CoPc@CNT-NH_2 in 0.2 M H_2SO_4 and 0.1 M $(NH_4)_2SO_4$ anolyte at 60 ℃ and 3 atm. (a) CO_2 flow rate set to 50.0 sccm before the reaction. (b) CO_2 flow rate reduced to 15.9 sccm during the reaction.

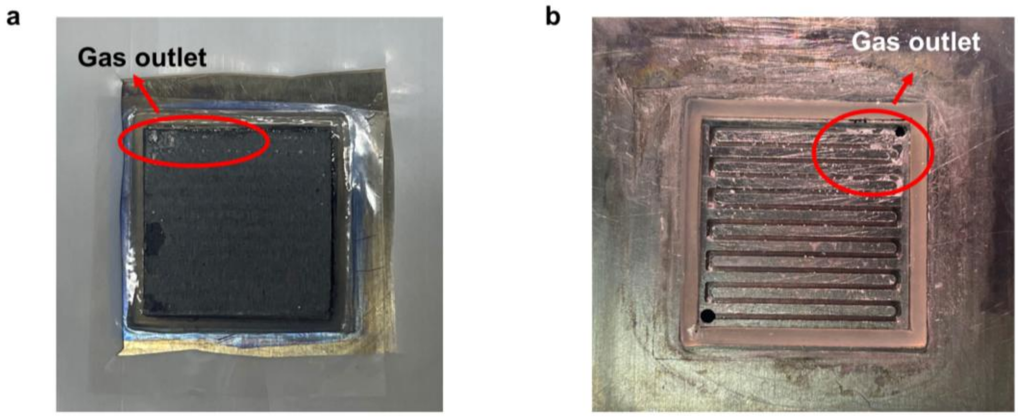

Figure S29. Distribution of salt particles observed after disassembling the reactor post-reaction on CoPc@CNT-NH_2 in 0.2 M H_2SO_4 and 0.1 M $(NH_4)_2SO_4$ anolyte at 60 ℃ and 3 atm. (a) Salt particles deposited on the back of the gas diffusion electrode. (b) Salt particles accumulated in the cathode flow field.

Table S1. Parameters and coefficients used in the GMPNP simulations.

Parameter	Value	Unit
D_{H^+}	9.311×10^{-9}[26]	$m^2 \cdot s^{-1}$
$D_{NH_4{}^+}$	1.908×10^{-9}[26]	$m^2 \cdot s^{-1}$
D_{K^+}	1.957×10^{-9}[26]	$m^2 \cdot s^{-1}$
D_{OH^-}	5.273×10^{-9}[26]	$m^2 \cdot s^{-1}$
$D_{SO_4{}^{2-}}$	1.065×10^{-9}[27]	$m^2 \cdot s^{-1}$
z_{H^+}	$+1$	-
$z_{NH_4{}^+}$	$+1$	-
z_{K^+}	$+1$	-
z_{OH^-}	-1	-
$z_{SO_4{}^{2-}}$	-2	-
a_{H^+}	5.60×10^{-10}[26]	m
$a_{NH_4{}^+}$	6.62×10^{-10}[26]	m
a_{K^+}	6.62×10^{-10}[26]	m
a_{OH^-}	6.00×10^{-10}[26]	m
$a_{SO_4{}^{2-}}$	8.00×10^{-10}[27]	m
F	96485	$C \cdot mol^{-1}$
R	8.314	$J \cdot mol^{-1} \cdot K^{-1}$
T	298.15	K
N_A	6.021×10^{23}	mol^{-1}
ω	900	s^{-1}
v	1.0×10^{-6}[28]	$m^2 \cdot s^{-1}$
K_w	1.0×10^{-14}[26]	M^2
k_{w1}	0.0204[26]	$mol \cdot m^{-3} \cdot s^{-1}$
k_{w2}	2.4×10^{6}[26]	$mol^{-1} \cdot m^3 \cdot s^{-1}$
ε_0	8.8542×10^{-12}[26]	$F \cdot m^{-1}$
ε_r	80.1[26]	-

Table S2. Performance comparison of acidic CO_2 electrolysis in MEA electrolyzers at current density of 100 mA cm^{-2}.

Catalyst	Anolyte	Cell voltage[a] (V)	Stability[b] (h)	CO_2 selectivity[c]	Energy efficiency	Ref.
**CoPc@CNT-NH_2**	**0.2 M H_2SO_4 & 0.1 M $(NH_4)_2SO_4$**	**2.84**	**110**	**86%**	**40.6%**	**This work**
Ag/PDDA-GO	0.01 M H_2SO_4	3.6	22	70%	26.1%	Ref. 29[29]
Ni-N-C	0.5M K_2SO_4 & H_2SO_4 (pH 0.5)	3.1	20	92%	39.8%	Ref. 30[30]
Ag	0.01 M H_2SO_4 & 0.01 M Cs_2SO_4	3.6	12	60%	22.3%	Ref. 31[31]
Cu/PCRL	0.01 M H_2SO_4	4	9	78%	26.1%	Ref. 32[32]

Notes:

a: Average cell voltage during stability test

b: Duration maintaining initial selectivity

c: Average CO_2 reduction selectivity during stability test

Table S3. Performance comparison of acidic and neutral CO_2 electrolysis in MEAs at current density of 100 mA cm^{-2}.

Catalyst	Anolyte	Cell voltage (V)	Stability (h)	CO_2 selectivity	SPCE	Energy efficiency	Ref.
**CoPc@CNT-NH_2**	**0.2 M H_2SO_4 & 0.1 M $(NH_4)_2SO_4$**	**2.84**	**110**	**86%**	**94%**	**40.6%**	**This work**
Ag	0.01 M $KHCO_3$	3.5	1000	98%	3.4%	37.5%	Hao’s work[24]

Table S4. Key parameters of acidic and neutral CO_2 electrolysis in MEAs at current density of 100 mA cm^{-2} for the estimation of energy consumption and operating cost under the idealized and quasi-realistic cases.

**Current density (mA cm^{-2})**	**Ideal FE (%)**	**Ideal SPCE (%)**	**Quasi-realistic FE (%)**	**Quasi-realistic SPCE (%)**	**Cell voltage (V)**	**Temperature (℃)**	**Ref.**
**100**	**86**	**94**	**68**	**94**	**2.84**	**60**	**This work**
100	98	49	80	44	3.5	25	Hao's work[24]

Table S5. Comparison of cobalt loss for CoPc@CNT-NH_2 and CoPc@CNT catalysts during electrolysis.

Catalyst	Effective catalyst loading (mg)	Initial Co content (ωt%)	Time (h)	Final Co content (ωt%)	Co loss (μg)	Co loss rate (μg h^{-1})
CoPc@CNT-NH_2	2	0.25	110	0.08	3.4	0.031
CoPc@CNT	2	0.23	20	0.07	3.2	0.160

Table S6. CO_2 flow rates measured using a soap film flowmeter at initial and in-reaction stages.

Stage	Displayed flow rate (sccm)	Measured flow rate (sccm)			Average measured flow rate (sccm)	Standard deviation (sccm)
Initial	50.0	49.91	50.68	49.97	50.19	0.43
In-reaction	15.9	15.89	15.95	15.90	15.91	0.03

References

1. Qin H-G, Li F-Z, Du Y-F *et al.* Quantitative understanding of cation effects on the electrochemical reduction of CO_2 and H^+ in acidic solution. *ACS Catal* 2023;**13**:916–26.

2. Kresse G, Furthmüller J. Efficiency of ab-initio total energy calculations for metals and semiconductors using a plane-wave basis set. *Comput Mater Sci* 1996;**6**:15–50.

3. Hammer B, Hansen LB, Nørskov JK. Improved adsorption energetics within density-functional theory using revised Perdew-Burke-Ernzerhof functionals. *Phys Rev B* 1999;**59**:7413–21.

4. Kresse G, Joubert D. From ultrasoft pseudopotentials to the projector augmented-wave method. *Phys Rev B* 1999;**59**:1758–75.

5. Mathew K, Sundararaman R, Letchworth-Weaver K *et al.* Implicit solvation model for density-functional study of nanocrystal surfaces and reaction pathways. *J Chem Phys* 2014;**140**:084106.

6. Resasco J, Chen LD, Clark E *et al.* Promoter effects of alkali metal cations on the electrochemical reduction of carbon dioxide. *J Am Chem Soc* 2017;**139**:11277–87.

7. Becke AD. Density-functional thermochemistry. III. The role of exact exchange. *J Chem Phys* 1993;**98**:5648–52.

8. Weigend F, Ahlrichs R. Balanced basis sets of split valence, triple zeta valence and quadruple zeta valence quality for H to Rn: Design and assessment of accuracy. *Phys Chem Chem Phys* 2005;**7**:3297–305.

9. Grimme S, Ehrlich S, Goerigk L. Effect of the damping function in dispersion corrected density functional theory. *J Comput Chem* 2011;**32**:1456–65.

10. Marenich AV, Cramer CJ, Truhlar DG. Universal Solvation Model Based on Solute Electron Density and on a Continuum Model of the Solvent Defined by the Bulk Dielectric Constant and Atomic Surface Tensions. *J Phys Chem B* 2009;**113**:6378–96.

11. Oh Y, Hu X. Organic molecules as mediators and catalysts for photocatalytic and electrocatalytic CO_2 reduction. *Chem Soc Rev* 2013;**42**:2253–61.

12. Li M, Irtem E, Iglesias van Montfort H-P *et al.* Energy comparison of sequential and integrated CO_2 capture and electrochemical conversion. *Nat Commun* 2022;**13**:5398.

13. Natural gas - monthly price - commodity prices - price charts, data, and news - IndexMundi.

14. Yue P, Kang Z, Fu Q *et al.* Life cycle and economic analysis of chemicals production via electrolytic (bi)carbonate and gaseous CO_2 conversion. *Appl Energy* 2021;**304**:117768.

15. Steegstra P, Ahlberg E. *In situ* pH measurements with hydrous iridium oxide in a rotating ring disc configuration. *J Electroanal Chem* 2012;**685**:1–7.

16. Yamanaka K. Anodically electrodeposited iridium oxide films (AEIROF) from alkaline solutions for electrochromic display devices. *Jpn J Appl Phys* 1989;**28**:632.

17. Zhang F, Co AC. Direct evidence of local pH change and the role of alkali cation during CO_2 electroreduction in aqueous media. *Angew Chem Int Ed* 2020;**59**:1674–81.

18. Monteiro MCO, Philips MF, Schouten KJP *et al.* Efficiency and selectivity of CO_2 reduction to CO on gold gas diffusion electrodes in acidic media. *Nat Commun* 2021;**12**:4943.

19. Garg S, Xu Q, Moss AB *et al.* How alkali cations affect salt precipitation and CO_2 electrolysis performance in membrane electrode assembly electrolyzers. *Energy Environ Sci* 2023;**16**:1631–43.

20. Monteiro MCO, Dattila F, Hagedoorn B *et al.* Absence of CO_2 electroreduction on copper, gold and silver electrodes without metal cations in solution. *Nat Catal* 2021;**4**:654–62.

21. Tüysüz H. Alkaline water electrolysis for green hydrogen production. *Acc Chem Res* 2024;**57**:558–67.

22. Jiang Y-C, Dong S-M, Liang Z *et al.* Holistic dynamic modeling and simulation of alkaline water electrolysis systems based on heat current method. *Energies* 2024;**17**:6202.

23. Scheepers F, Stähler M, Stähler A *et al.* Improving the efficiency of PEM electrolyzers through membrane-specific pressure optimization. *Energies* 2020;**13**:612.

24. Hao S, Elgazzar A, Ravi N *et al.* Improving the operational stability of electrochemical CO_2 reduction reaction via salt precipitation understanding and management. *Nat Energy* 2025;**10**:266–77.

25. Gu J, Liu S, Ni W *et al.* Modulating electric field distribution by alkali cations for CO_2 electroreduction in strongly acidic medium. *Nat Catal* 2022;**5**:268–76.

26. Bohra D, Chaudhry JH, Burdyny T *et al.* Modeling the electrical double layer to understand the reaction environment in a CO_2 electrocatalytic system. *Energy Environ Sci* 2019;**12**:3380–9.

27. Lide DR, Baysinger G, Chemistry S *et al.* *CRC Handbook of Chemistry and Physics*. CRC press, 2004.

28. Grozovski V, Vesztergom S, Láng GG *et al.* Electrochemical hydrogen evolution: H^+ or H_2O reduction? A rotating disk electrode study. *J Electrochem Soc* 2017;**164**:E3171–8.

29. Fan J, Pan B, Wu J *et al.* Immobilized tetraalkylammonium cations enable metal-free CO_2 electroreduction in acid and pure water. *Angew Chem Int Ed* 2024;**136**:e202317828.

30. Li H, Li H, Wei P *et al.* Tailoring acidic microenvironments for carbon-efficient CO_2 electrolysis over a Ni-N-C catalyst in a membrane electrode assembly electrolyzer. *Energy Environ Sci* 2023;**16**:1502–10.

31. Pan B, Fan J, Zhang J *et al.* Close to 90% single-pass conversion efficiency for CO_2 electroreduction in an acid-fed membrane electrode assembly. *ACS Energy Lett* 2022;**7**:4224–31.

32. O’Brien CP, Miao RK, Liu S *et al.* Single pass CO_2 conversion exceeding 85% in the electrosynthesis of multicarbon products via local CO_2 regeneration. *ACS Energy Lett* 2021;**6**:2952–9.
